# Selective Gas-Phase Ethylene Dimerization to 1‑Butene in a Scalable Metal–Organic Framework

**DOI:** 10.1021/jacs.5c21528

**Published:** 2026-02-17

**Authors:** Eric You, Xiao Zhang, Patrick Sarver, Mircea Dincă

**Affiliations:** † Department of Chemistry, 6740 Massachusetts Institute of Technology, 77 Massachusetts Avenue, Cambridge, Massachusetts 02139, United States; ‡ Department of Chemistry, Princeton University, Princeton, New Jersey 08544, United States

## Abstract

High-purity 1-butene is a crucial feedstock for the plastics industry and is currently produced by homogeneously catalyzed ethylene dimerization. Processes of this scale can benefit from heterogeneous catalysis, especially if reactants are delivered in the gas phase, to allow product recovery in flow. However, catalysts for gas-phase ethylene dimerization are exceedingly rare and generally show very low activity or reduced selectivity. Here, we report the use of a scalable, robust MOF catalyst (nickel-exchanged CFA-1, **Ni-CFA-1**) for the continuous gas-phase dimerization of ethylene to produce 1-butene. Operating under solvent-free conditions in a packed-bed reactor containing the MOF catalyst preactivated with a simple and straightforward approach, **MeNi-CFA-1** delivers excellent selectivity (96%), turnover frequencies greater than 800,000 mol ethylene·mol Ni^–1^·h^–1^, and total turnover numbers of 1.23 × 10^8^ mol ethylene·mol Ni^–1^, all exceeding the values observed for the commercial homogeneous catalyst. This flow process produces 49.4 kg 1-butene·g MOF^–1^ without requiring catalyst reactivation. The elimination of solvent allows a significantly higher concentration of ethylene near the Ni active sites, while the flow process drives away the butene product, thus suppressing undesired isomerization and oligomerization byproducts. Overall, this work highlights how MOFs can facilitate reactivity inconceivable for a molecular analogue and bridge the gap between molecular precision and industrial practicality, broadly illustrating the value of MOFs for the development of novel, selective, and scalable heterogeneous processes for the production of commodity chemicals.

## Graphical abstract

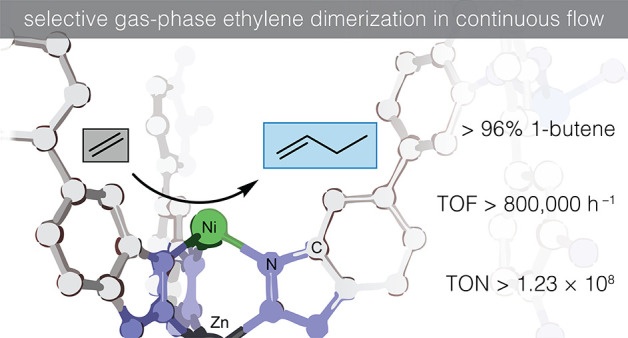

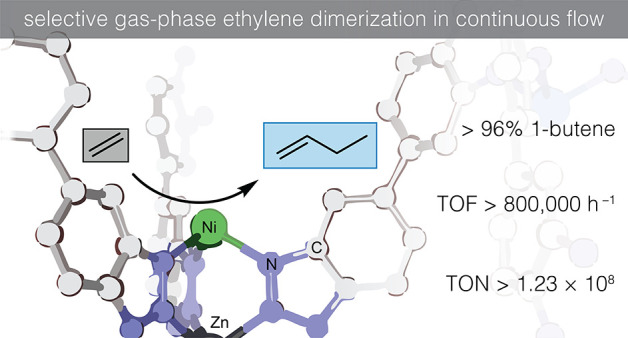

## Introduction

Global demand for linear low-density polyethylene (LLDPE), the dominant polymer in food packaging and preservation,^1^ exceeded 40 Mt in 2024 ^2^ and is expected to grow nearly 3-fold by 2040.^3^ This growing demand requires improved access to linear alpha olefins, the comonomers for LLDPE synthesis.^1^ Selective oligomerization of ethylene can conceivably generate alpha olefin products with perfect atom economy, but this approach suffers from inherent challenges of chain-length selectivity and product isomerization that often forms inseparable internal olefins.^4^ The most successful processes employ homogeneous catalysts, for which careful control of the metal and ligand structure provides the requisite steric and electronic environment to favor the desired terminal olefin products.^5^ For example, the titanium-based AlphaButol ethylene dimerization process remains one of the largest homogeneous catalytic industrial transformations, conducted on nearly a megaton scale annually (Figure 1a).^5,6^ Processes of this scale can benefit from heterogeneous catalysis, especially if reactants are delivered in the gas phase, to allow product recovery in flow.^7^ Indeed, developing a continuous, heterogeneous ethylene dimerization route to selectively generate 1-butene has been a long-standing challenge.^8–18^

**Figure 1:**
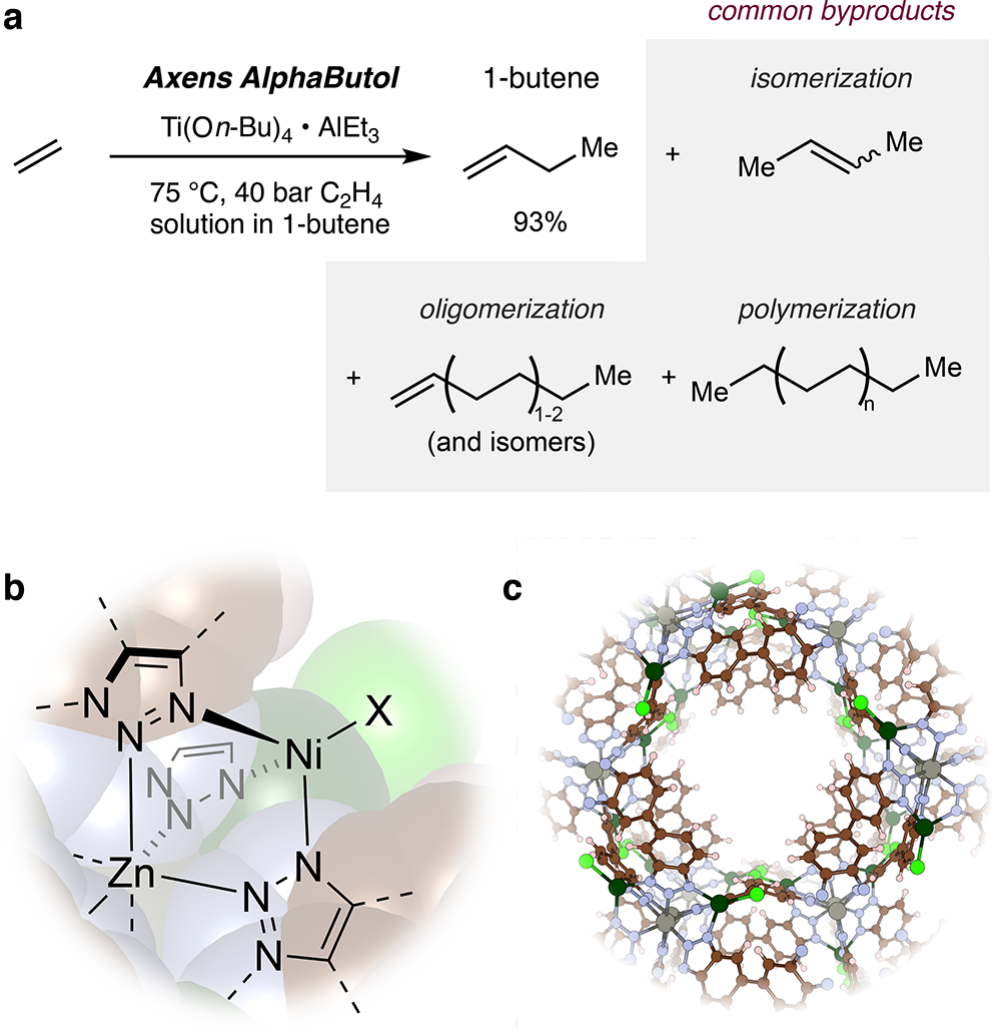
(a) Current state-of-the-art of the AlphaButol process; (b) active site of **Ni-CFA-1** for ethylene dimerization; (c) MOF structure of **Ni-CFA-1**.

Metal–organic frameworks (MOFs)—a class of porous, crystalline solids composed of metal clusters joined by organic linkers—combine many of the benefits of molecular homogeneous catalysts with the key advantages of heterogeneity.^7^ The principles of reticular chemistry enable precise modulation of the ligand sphere and pore structure, and environment surrounding catalytic active sites with comparable efficiency to molecular catalysts.^19^ MOF catalysts are thus uniquely suited to cases in which selectivity between related products demands careful control of catalyst structure, while at the same time the desired application necessitates continuous processing and catalyst recycling. Though constraints of pore diffusion limit the conceivable size of substrates amenable to MOF catalysis,^20^ the nature of large-scale processes that benefit most from catalyst heterogeneity—those that employ small, gaseous substrates at high pressures—indicates that MOF catalysts might be privileged for such large-scale feedstock upconversions.

Although MOFs have been shown to catalyze ethylene dimerization,^21^ none to date demonstrate the combination of activity,^7,8^ selectivity,^13,14^ and stability^17,22–24^ required to enable practical, continuous gas-phase dimerization (Figure 5 and Table S3). Current processes operate in the solution phase because a constant resupply of catalyst and a relatively high concentration of cocatalyst, both molecular species, are required for sustained catalysis. Developing a competitive gas-phase heterogeneous process for this transformation would thus necessarily have to address two fundamental challenges: obviate the need for constant catalyst resupply and alleviate the requirement of a solubilized cocatalyst. Although a catalyst driving such a gas-phase process should maintain high selectivity with minimal production of polyethylene and high activity at relevant conversion rates, it would greatly enhance overall process mass intensity, which is suppressed in the liquid phase.

## Results and Discussion

To this end, we looked to transform recent initial successes with a nickel-exchanged bis-bibenzotriazole framework [Ni_*x*_Zn_5–*x*_(OAc)_4_(bibta)_3_], H_2_bibta = 1H,1H′-5,5′-bibenzo­[d]­[1,2,3]­triazole, **Ni-CFA-1** (Figure 1b,c).^9^ Although previous reports with **Ni-CFA-1** emphasized its excellent selectivity, activity, and stability for 1-butene production, reaction studies were limited to dilute catalyst slurries and batch reactors that employed a large excess of alkylaluminum activator. To translate these results to a gas-phase continuous process, we envisioned that this framework might survive preactivation with MMAO-12 and the subsequent removal of solvent to generate the active catalyst as a solid.

CFA-1 and Ni-exchanged variants with Ni contents ranging from 0.4% to 60% were synthesized by adapting published procedures.^25^ Treating **Ni**(2%)**-CFA-1** with a solution of MMAO-12 (MMAO = modified methylaluminoxane, 1000 equiv Al/Ni) in toluene, followed by concentrating the reaction mixture to a solid *in vacuo* and pressurizing with ethylene, afforded significant formation of butenes in a batch reactor. Evidently, this solid contains a preactivated form of **Ni**(2%)**-CFA-1** that is competent for ethylene dimerization, showing a TOF of 78,000 mol C_2_H_4_·mol Ni^–1^·h^–1^. Treatment of pure **Ni**(2%)**-CFA-1** with the solution of MMAO-12 likely yields a Ni-methyl species, implicated previously in liquid-phase catalysis.^9,26^ We denote this prealkylated catalyst **MeNi**(2%)**-CFA-1** (Figure 2a,b). Importantly, exposing **Ni**(2%)**-CFA-1** to 50 bar of ethylene at ambient temperature did not produce any butenes, consistent with the reported need for an alkylaluminum cocatalyst. Similarly, MMAO-12 in the absence of **Ni**(2%)**-CFA-1** is also not a competent catalyst for ethylene dimerization under these conditions, even though alkylaluminums have been known to catalyze ethylene oligomerization at high ethylene pressures and temperatures.^27^

**Figure 2:**
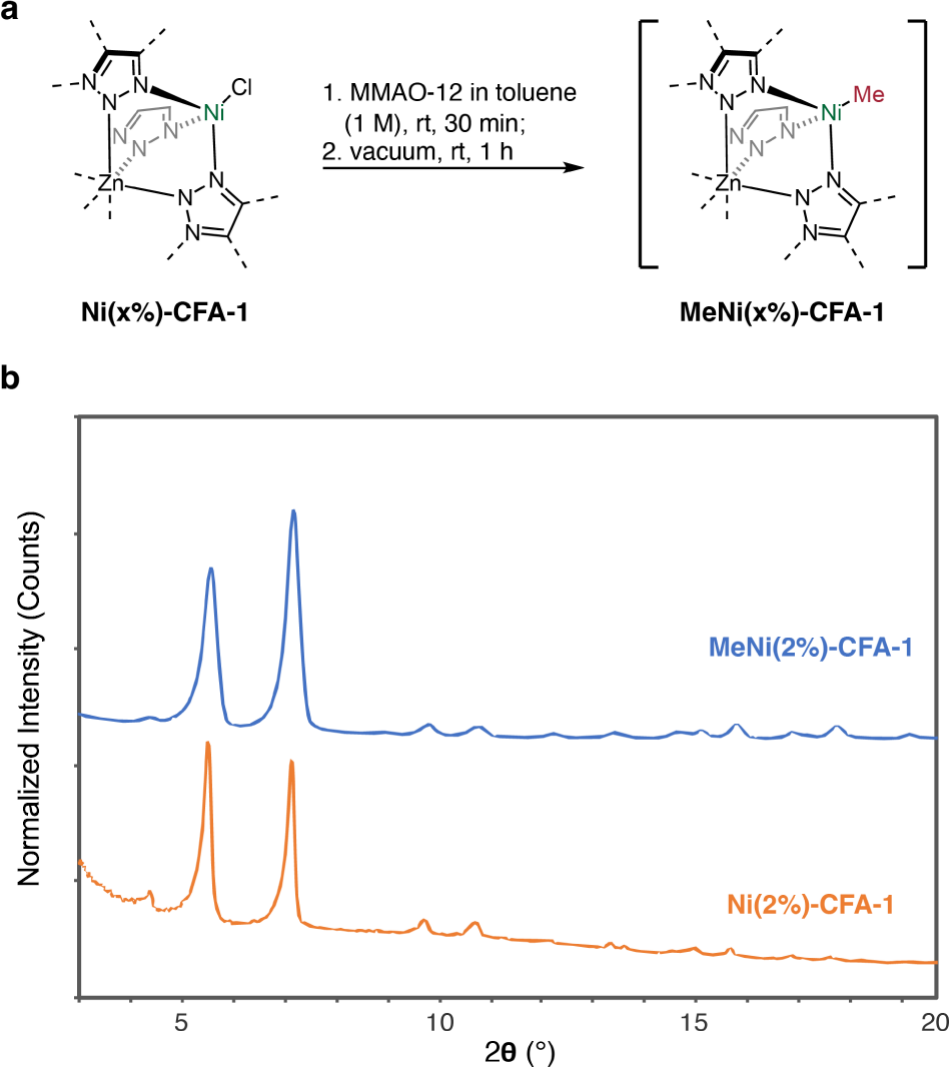
(a) Preactivation of **Ni-CFA-1** and removal of solvent; (b) PXRD patterns of the **Ni**(2%)**-CFA-1** (bottom) and **MeNi**(2%)**-CFA-1** (top).

Although the first experiments with **MeNi**(2%)**-CFA-1** supported the general feasibility of our approach, the catalyst did show reduced selectivity for C4 products, achieving only 93.4%, and for 1-butene versus 2-butenes: 52.2% vs 47.8%, respectively. With the solid catalyst in hand, we were nevertheless excited about the possibility of translating this process to a flow system. Indeed, we assigned the poor selectivity to a buildup of liquid 1-butene in the batch reactor, which essentially forms a suspension of the catalyst in 1-butene. This increases the concentration of butene relative to ethylene, favoring the isomerization to 2-butenes and the formation of longer oligomers (Figure 3a).

**Figure 3:**
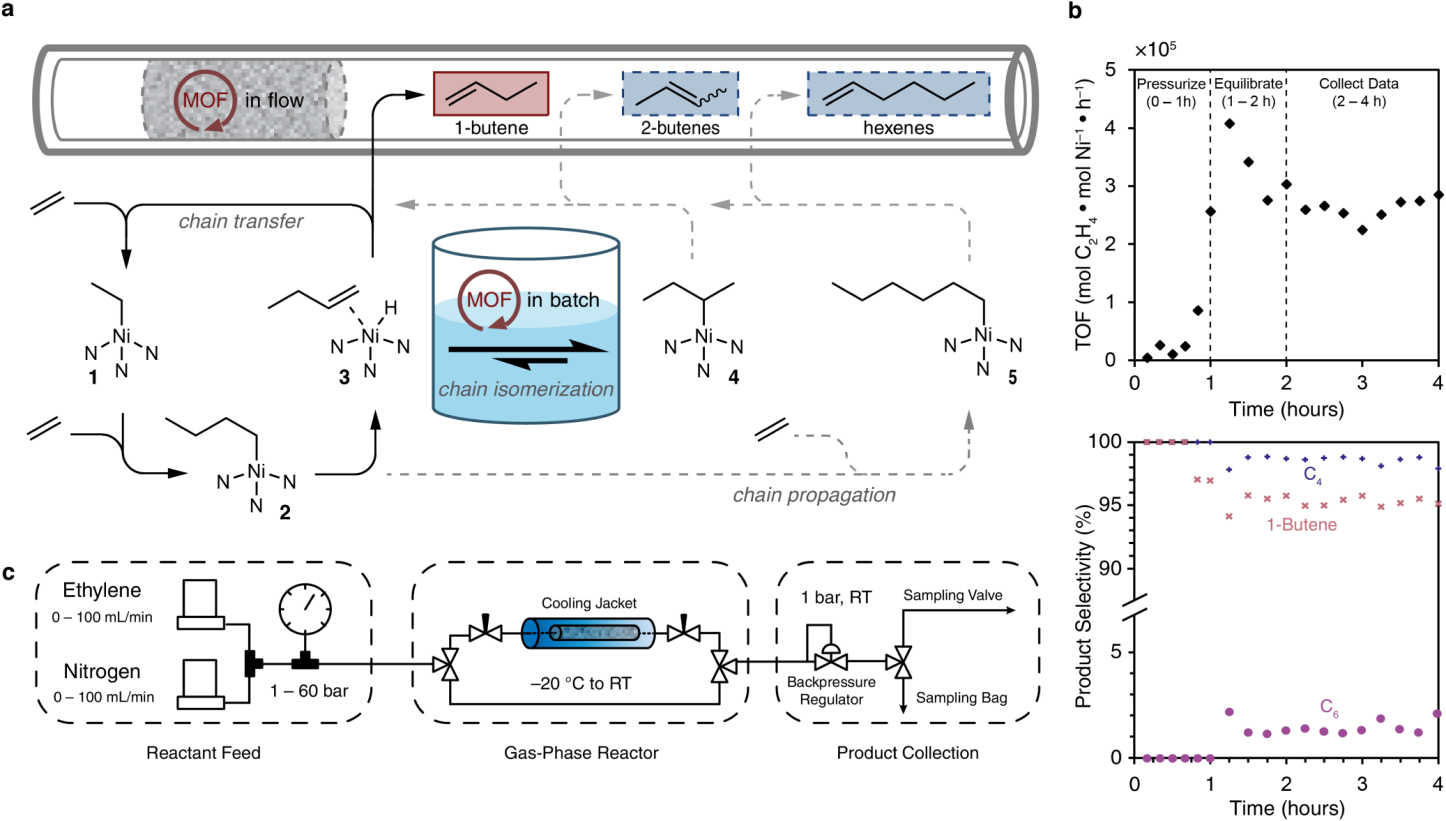
(a) Illustration of key mechanistic considerations in dimerization between operating in flow and in batch; (b) flow reaction time diagram; (c) flow reactor setup schematic.

To avoid butene buildup, we designed a reactor to flow ethylene through a packed bed of **MeNi-CFA-1**. To further minimize exposure of the catalyst to 1-butene, we diluted the catalyst bed with inert silicon carbide. The reactor was also fitted with a thermocouple inserted just below the catalyst bed and equipped with a temperature-controlled jacket that allowed reaction temperatures ranging from −20 °C to room temperature (Figures 3c and S17).

Upon carrying out the gas-phase dimerization in flow with **MeNi**(1.5%)**-CFA-1** at 50 bar, 23 °C (Figure 4d and Table S2, entry 1), C_4_ product selectivity increased to 97.4% and overall 1-butene selectivity greatly improved to 90.0%, validating the utility of a flow reactor for gas-phase catalysis. Surprisingly, however, the activity of **MeNi**(1.5%)**-CFA-1** on a per-nickel basis was much lower than expected, at only 9500 mol ethylene·mol Ni^–1^·h^–1^ (Figure 4a). This suggested that not all Ni atoms were active in the catalysis. In other words, due to the fast reaction rate, supplying ethylene at this pressure does not fully utilize all Ni active sites. To verify this, we further decreased the Ni content of the catalyst to 0.4% to decrease the active site density within the MOF. Indeed, **MeNi**(0.4%)**-CFA-1** shows significantly higher per-nickel activity of 56,000 mol C_2_H_4_·mol Ni^–1^·h^–1^ with an even slightly increased overall selectivity for 1-butene of 91%. Conversely, **MeNi**(60%)**-CFA-1**, a catalyst with much higher Ni loading, showed a low activity of 7100 mol C_2_H_4_·mol Ni^–1^·h^–1^ and greatly reduced selectivity of 60.3% for 1-butene, suggesting that in the absence of sufficient ethylene substrate, excess active Ni sites start utilizing butene products as substrates. Comparison of the PXRD patterns of **MeNi-CFA-1** and pristine **Ni-CFA-1** with three different nickel loadings (Figure S4) reveals that the crystallinity of **MeNi**(1.5%)**-CFA-1** and **MeNi**(0.4%)**-CFA-1** is maintained, whereas the crystallinity of **MeNi**(60%)**-CFA-1** is lost. Consistently, N_2_ adsorption isotherms of **MeNi**(1.5%)**-CFA-1** and **MeNi**(0.4%)**-CFA-1** (Figure S8 and S6) demonstrate almost no loss in surface area after MMAO activation compared to the pristine **Ni-CFA-1** (from 2111 m^2^/g to 2104 m^2^/g, and from 1802 m^2^/g to 1639 m^2^/g, respectively), while **MeNi**(60%)**-CFA-1** (Figure S7) displays a markedly reduced surface area of 553 m^2^/g compared to 2212 m^2^/g for pristine **MeNi**(60%)**-CFA-1**. This substantial loss of porosity and crystallinity of **MeNi**(60%)**-CFA-1** might also contribute to the significantly lower ethylene dimerization activity compared to **MeNi**(1.5%)**-CFA-1** and **MeNi**(0.4%)**-CFA-1**.

**Figure 4:**
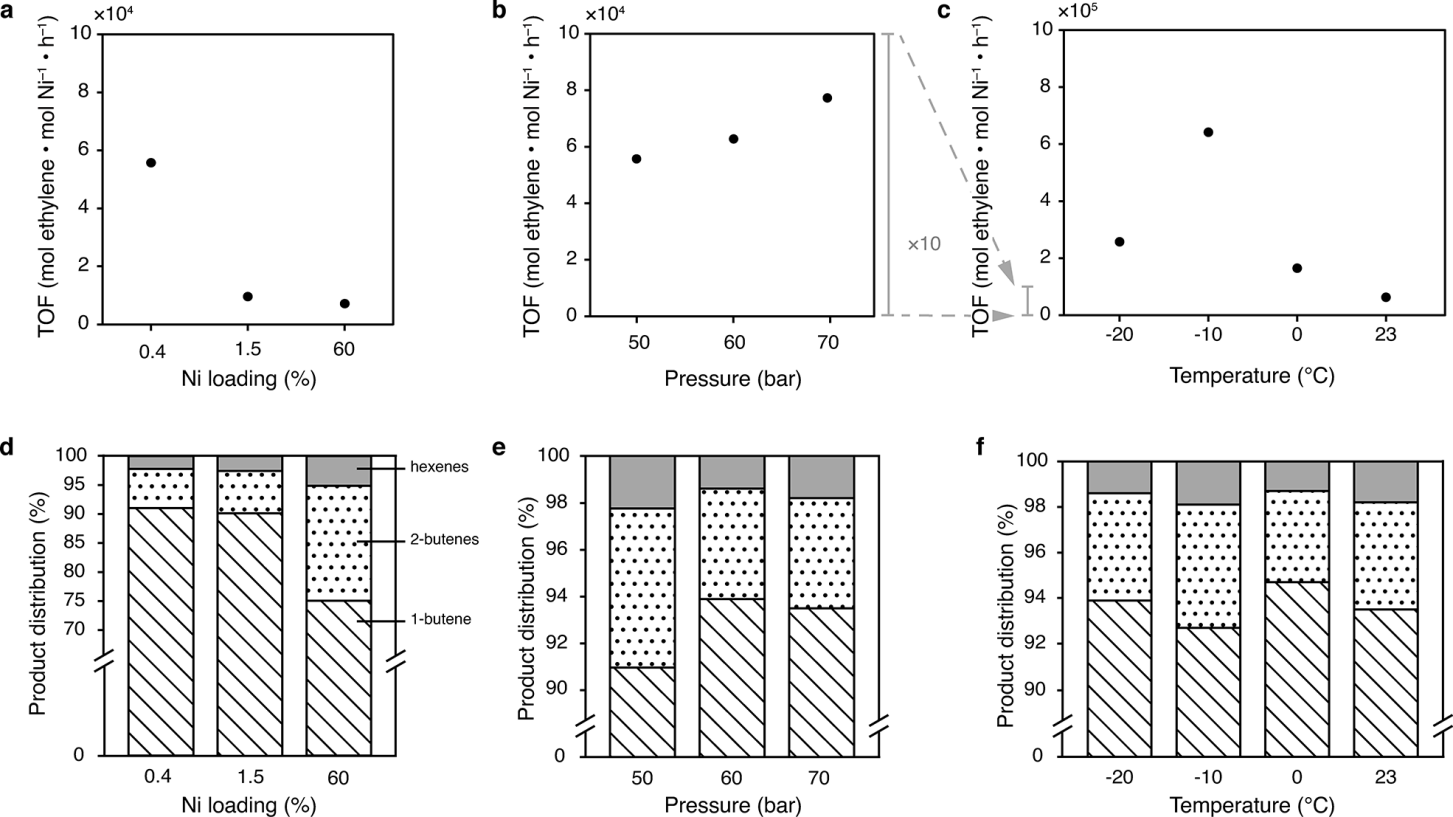
(a) Effects of Ni loading on activity of ethylene dimerization at 23 °C, 50 bar; (b) effects of pressure on activity of ethylene dimerization with **MeNi**(0.4%)**-CFA-1** at 23 °C; (c) effects of temperature on activity of ethylene dimerization with **MeNi**(0.4%)**-CFA-1** at 60 bar; (d) effects of Ni loading on selectivity of ethylene dimerization at 23 °C, 50 bar; (e) effects of pressure on selectivity of ethylene dimerization with **MeNi**(0.4%)**-CFA-1** at 23 °C; (f) effects of temperature on selectivity of ethylene dimerization with **MeNi**(0.4%)**-CFA-1** at 60 bar.

Having identified **MeNi**(0.4%)**-CFA-1** as a selective catalyst with competitive activity, we took advantage of the flow catalytic system to further optimize reaction outcomes by changing the pressure and temperature along the phase diagram of ethylene. To this end, we screened several pressure and temperature conditions through 4 h experiments (Table S2), from which the weighted average of TOF and selectivity were recorded between time points at 2 and 4 h to allow quasi-steady-state measurements (Figure 3b). Thus, increasing pressure monotonically leads to higher activity from 55,700 mol ethylene ·mol Ni^–1^·h^–1^ at 50 bar to 77,300 mol C_2_H_4_·mol Ni^–1^·h^–1^ at 70 bar, with a similar jump in 1-butene selectivity from 91.0% at 50 bar to 93.5% at 70 bar (Figure 4b,e). Although a pressure of 70 bar led to the highest activity, further optimization was continued at 60 bar, due to the difficulty of maintaining high inlet pressure. On the temperature axis, reactions run at −10 °C yielded the highest TOF of 640,000 mol C_2_H_4_·mol Ni^–1^·h^–1^, a full order of magnitude larger than at room temperature (Figure 4c). Notably, working at such reduced temperatures in batch reactions leads to much lower activity, presumably owing to slower ethylene diffusion through the solvent.^9,15^ Under gas-phase flow conditions, the increased ethylene concentration likely balances this effect, even as the dynamic viscosity of ethylene at −10 °C more than triples relative to the value at 23 °C (59 vs 17 μPa·s).^28^ The increase in rate with decreasing temperature also exceeds what would be expected based on just an increase in ethylene concentration of ∼2.9 (Figure S19) and could indicate the presence of liquid ethylene within pores, which has been shown before to increase catalyst activity and selectivity, as well as lead to deviations from a thermodynamically ideal system.^22,29^

At −10 °C, the conversion is also much higher than at 0 °C: 20% and 5.6%, respectively. However, the overall selectivity for 1-butene at the lower temperature slightly decreased to 92.7% from 93.5% at 0 °C (Figure 4f). Nevertheless, we could recover higher selectivity by lowering the overall mass loading. The final optimized activity for **MeNi**(0.4%)**-CFA-1** at −10 °C and 60 bar of ethylene was 610,000 mol C_2_H_4_·mol Ni^–1^·h^–1^ with an overall 1-butene selectivity of 95.3% (Table S2, entry 11). These values compare favorably not just to previous homogeneous and heterogeneous catalysts in liquid or gas phase, but stand alone as a combination of activity and selectivity that both surpass the commercial AlphaButol catalyst (Figure 5).^8–11^ Such a combination of activity and selectivity makes **MeNi**(0.4%)**-CFA-1** the first catalyst in a continuous gas-phase flow process that exceeds the activity and selectivity of AlphaButol, though recent works, such as Ni-MCM-41 ^29^ and UiO-66-NHPiPr_2_–NiCl_2_,^30^ have demonstrated high activity of 38,900 mol ethylene·mol Ni^–1^·h^–1^ and excellent 1-butene selectivity >99%, respectively. Given the improvement in optimized conditions from slurry to gas phase, we would also like to highlight Diimine-Ni@PCN-701,^31^ 15Ni-ZIF-L,^10^ Ni-(Fe)­MIL-101,^14^ and Ni-ZIF-8 ^11^ as catalysts that could potentially benefit from further study in gas-phase ethylene dimerization, following our simple strategy of preactivation, as they also use alkylaluminum activators.

**Figure 5:**
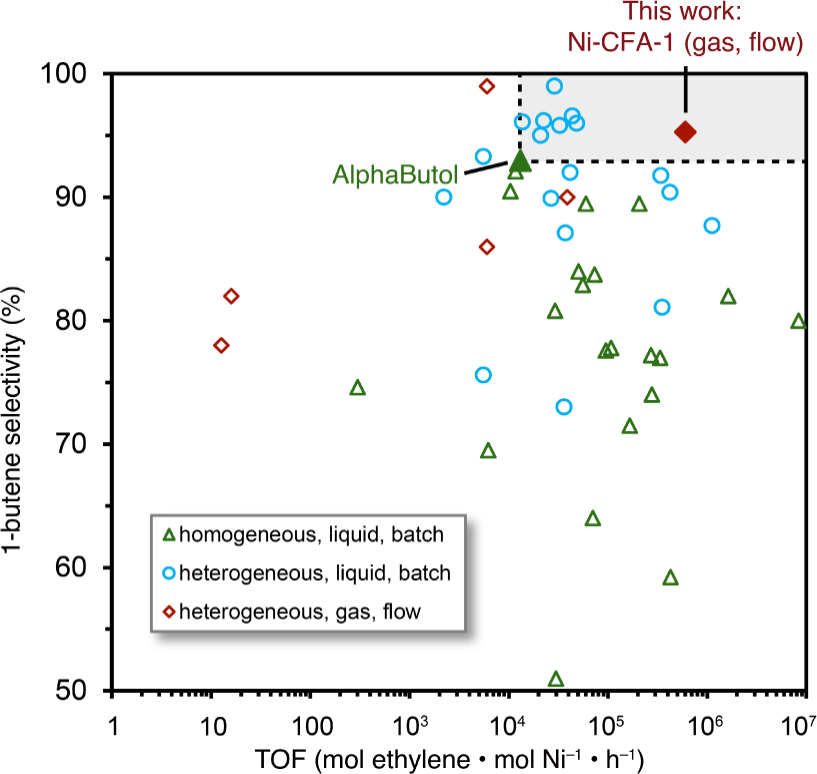
Comparison of the catalytic performance (TOF and 1-butene selectivity) of **Ni-CFA-1** in a gas-phase reaction with previously reported Ni-based catalysts (SI, Table S3).

This observation that selectivity increases with decreasing conversion prompted us to further investigate product distribution across a range of conversions from 2% to 27%. Using selectivity data for 1-butene, 2-butenes, and hexenes obtained from optimization experiments run with **MeNi**(0.4%)**-CFA-1** at 60 bar and −10 °C (Table S2, Entries 10 and 11), we constructed and fitted a kinetic model based on the Cossee–Arlman mechanism. In our model (Figure 6b), we hypothesized that the observed product selectivity can be described in two steps: an initial step selectively produces 1-butene (k_A1_), compared to 2-butenes (k_A2_) and hexenes (k_A3_), and a subsequent step that isomerizes 1-butene to form 2-butenes (k_B1_) or oligomerizes 1-butene (k_B2_) and 2-butenes (k_B3_) to form hexenes. The rate constants shown are defined relative to the overall first-order rate constant k_D_ = 1 (full derivation in the Supporting Information, Section S8). Indeed, fitting such a model proved quite successful, with a root-mean-square error of just 0.0179 across 1257 data points (Figure 6a). An initial catalytic turnover selectively produces 1-butene (k_A1_ = 0.9778 ≫ k_A2_, k_A3_), relative to 2-butenes (k_A2_), and hexenes (k_A3_). However, 1-butene builds up with increasing conversion and isomerization of 1-butene to 2-butenes becomes competitive with productive chain termination (k_B1,f_ ≈ k_A1_), while oligomerization to hexenes (k_B2_ < k_A1_) is disfavored. More importantly, the relative rate constants obtained from fitting the model permitted us to better understand the change from batch to flow. At the limit of extremely low conversions, the selectivity for 1-butene approaches a maximum of 97.8% selectivity by weight, indicated by our value of k_A1_ 2 orders of magnitude larger than k_A2_, k_A3_. Such high single-site selectivity for 1-butene agrees with past computational and kinetics studies of **Ni-CFA-1** in slurry batch reactors.^26,32^ Moreover, such a high selectivity for 1-butene exceeds the highest selectivity (96.1%) ever observed in slurry batch reactors under optimized conditions, validating the use of a flow reactor to perform gas-phase ethylene dimerization. As conversion increases and 1-butene concentration increases, secondary adsorption and isomerization of 1-butene to form 2-butenes (k_B1_) must be considered, where the relative rate of isomerization k_B1_ = 0.87264 indicates that isomerization is kinetically competitive with the initial rate of ethylene dimerization. In a gas-phase batch reactor, such buildup is deleterious. Nevertheless, our reactivity shows that in flow, conversion of up to 20% may be achieved while maintaining 1-butene selectivity greater than 93%.

**Figure 6:**
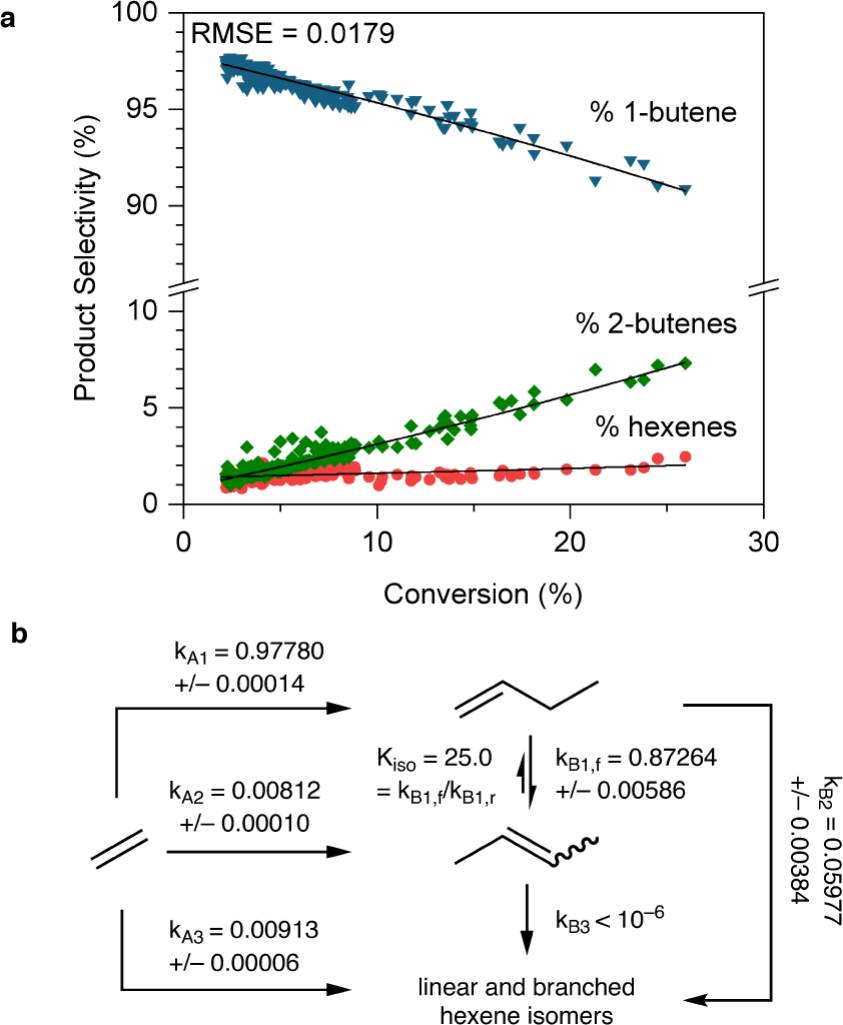
(a) Changes in product selectivity with increasing conversion using data from optimization experiments (Table S2, entries 10–11) were fitted to a kinetic model based on the Cossee–Arlman mechanism for ethylene dimerization; (b) kinetic model that describes the observed product selectivity in two steps: an initial step selectively produces 1-butene (k_A1_), compared to 2-butenes (k_A2_) and hexenes (k_A3_); and a subsequent step that isomerizes 1-butene to form 2-butenes (k_B1_) or oligomerizes 1-butene (k_B2_) and 2-butenes (k_B3_) to form hexenes. Full derivation is given in the Supporting Information, Section S8.

Having identified optimized conditions for ethylene dimerization, we assessed the overall lifetime and stability of the catalyst at 60 bar and −10 °C. **MeNi**(0.4%)**-CFA-1** exhibits exceptional activity and stability over the course of the first 16 h, maintaining an average activity of approximately 600,000 mol C_2_H_4_·mol Ni^–1^·h^–1^ and an overall 1-butene selectivity of 96% (Figure 7a). After 7 days, the activity of **MeNi**(0.4%)**-CFA-1** decays to an average of ∼200,000 mol C_2_H_4_·mol Ni^–1^·h^–1^. During the same period, the overall selectivity for 1-butene actually increased to ∼98%, consistent with a decrease in conversion to 2% (Figure 7b). Remarkably, this high activity and long lifetime of the catalyst cause a significant drop in pressure in the ethylene tanks available to academic laboratories.

**Figure 7:**
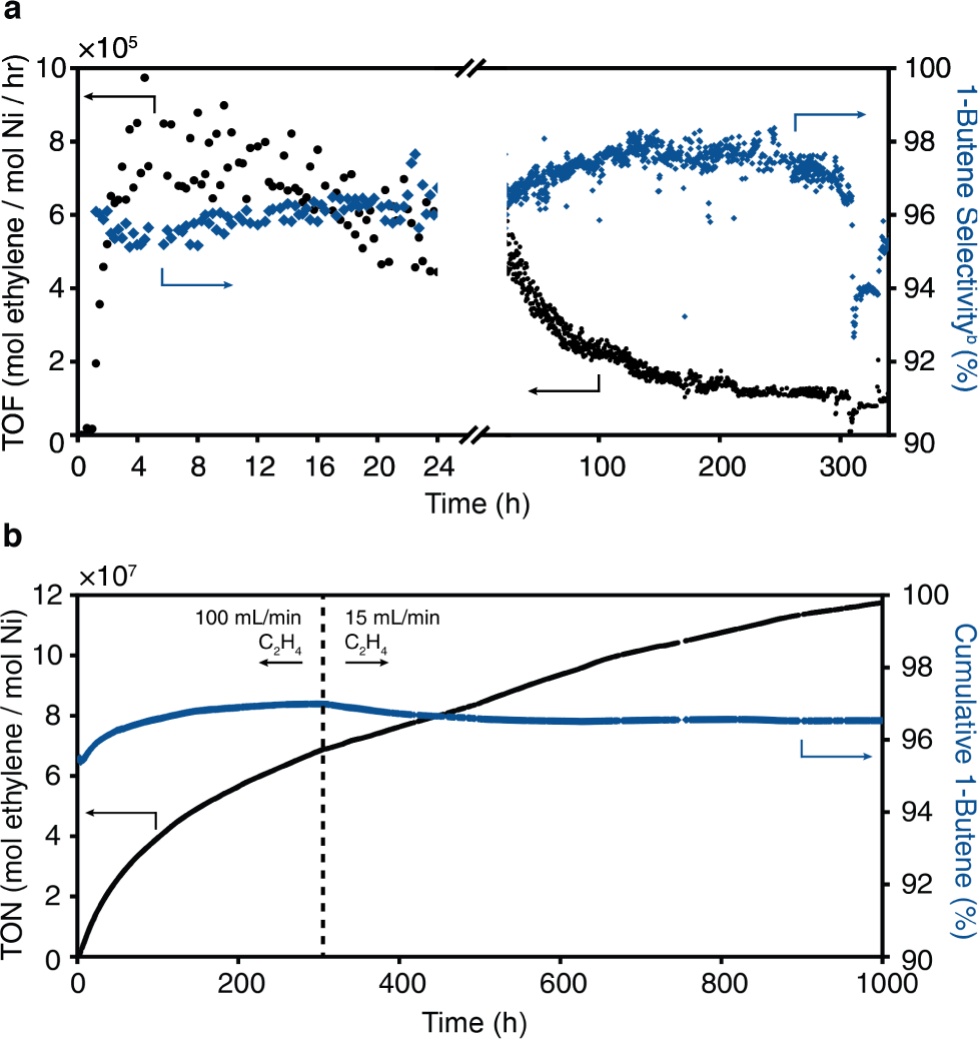
(a) Initial activity and selectivity of 2 mg **MeNi**(0.4%)**-CFA-1** through ramp-up pressurization to 60 bar, −10 °C demonstrating high activity and selectivity beyond the 2 to 4 h window, followed by subsequent decline in activity and increase in selectivity with 1-butene selectivity over 2 weeks; (b) cumulative turnover numbers and 1-butene selectivity among products over 2 months indicating record stability, activity, and selectivity among catalysts.

After 2 weeks of continuous operation, maintaining an inlet pressure of 60 bar required reducing the flow rate of ethylene from 100 to 15 mL/min, with an increase in conversion resulting in lower 1-butene selectivity. Although the activity does continue to decrease over time, **MeNi**(0.4%)**-CFA-1** maintains an average turnover frequency of 90,000 mol ethylene·mol Ni^–1^·h^–1^ over 6 weeks, without the need for additional cocatalyst, reactivation, or any other user input. After two months, the productivity of the catalyst is 1.23 × 10^8^ mol ethylene·mol Ni^–1^, or 49.4 kg 1-butene per g of MOF catalyst, far exceeding the 1.3 kg of 1-butene per g of catalyst in the AlphaButol process. To the best of our knowledge, no other heterogeneous or homogeneous catalyst has shown such sustained performance beyond a week, let alone one month—a technologically significant demonstration that advances MOFs for large-scale commodity chemical production (Figure S32).^33^ More importantly, the long-term stability of **MeNi**(0.4%)**-CFA-1** stands out among ethylene dimerization catalysts, which typically require large excesses of alkylaluminum to achieve high activity by continuous reactivation.^9^ The results here demonstrate that the simple strategy of preactivation can make MOFs selective, active, and stable gas-phase catalysts for the production of commodity chemicals.

## Conclusion

The foregoing results demonstrate the considerable potential for using MOFs under scalable configurations to catalyze industrially significant reactions. By mitigating the need for solvent and continual reactivation, this technology represents a step forward in improving sustainability without sacrificing catalyst properties. Indeed, the improved activity and selectivity of **Ni-CFA-1** in flow demonstrate the power of utilizing MOFs for heterogeneous solid–gas reactions. We anticipate that exploring further reactivity at the metal nodes of MOFs under gas-phase conditions will lead to important new applications for these highly tunable materials in heterogeneous catalysis.

## Supplementary Material



## Notes

Synthetic procedures, PXRD patterns, N_2_ isotherms, SEM images, design and construction of packed bed reactor, gas-phase ethylene dimerization experiments, gas chromatography chromatograms, optimization tables, and summary of homo- and heterogeneous catalysts for ethylene dimerization (PDF)

## References

[1] Zimmermann, H. ; Walzl, R. Ethylene. In Ullmann’s Encyclopedia of Industrial Chemistry; John Wiley & Sons, Ltd, 2009.

[2] Ranjan, A. J. ; Nandi, R. Linear Low-Density Polyethylene (LLDPE) Market Supply | 2032. Claight Corporation (Expert Market Research). https://www.expertmarketresearch.com/industry-statistics/linear-low-density-polyethylene-lldpe-market (accessed 27 August 2025).

[3] Richardson, J. The pandemic, climate change, plastic waste and the great divide: the world in 2025. Asian Chemical Connections. https://www.icis.com/asian-chemical-connections/2021/06/the-pandemic-climate-change-plastic-waste-and-the-great-divide-the-world-in-2025/ (accessed 2024 March 28).

[4] Speiser F., Braunstein P., Saussine L. (2005). Catalytic Ethylene Dimerization and
Oligomerization: Recent Developments with Nickel Complexes Containing
P, N-Chelating Ligands. Acc. Chem. Res..

[5] Bryliakov K.
P., Antonov A. A. (2018). Recent
Progress of Transition Metal Based Catalysts
for the Selective Dimerization of Ethylene. J. Organomet. Chem..

[6] Oligomerization | Axens https://www.axens.net/markets/petrochemicals/oligomerization (accessed 03 January 2024).

[7] Jimenez-Gonzalez C., Ponder C. S., Broxterman Q. B., Manley J. B. (2011). Using the Right
Green Yardstick: Why Process Mass Intensity Is Used in the Pharmaceutical
Industry To Drive More Sustainable Processes. Org. Process Res. Dev..

[8] Chen C., Meng L., Alalouni M. R., Dong X., Wu Z.-P., Zuo S., Zhang H. (2023). Ultra-Highly Active Ni-Doped MOF-5 Heterogeneous Catalysts
for Ethylene Dimerization. Small.

[9] Metzger E. D., Comito R. J., Wu Z., Zhang G., Dubey R. C., Xu W., Miller J. T., Dincă M. (2019). Highly Selective Heterogeneous Ethylene
Dimerization with a Scalable and Chemically Robust MOF Catalyst. ACS Sustainable Chem. Eng..

[10] Chen C., Alalouni M. R., Xiao P., Li G., Pan T., Shen J., Cheng Q., Dong X. (2022). Ni-Loaded
2D Zeolitic
Imidazolate Framework as a Heterogeneous Catalyst with Highly Activity
for Ethylene Dimerization. Ind. Eng. Chem. Res..

[11] Chen C., Alalouni M. R., Dong X., Cao Z., Cheng Q., Zheng L., Meng L., Guan C., Liu L., Abou-Hamad E., Wang J., Shi Z., Huang K.-W., Cavallo L., Han Y. (2021). Highly Active Heterogeneous Catalyst
for Ethylene Dimerization Prepared by Selectively Doping Ni on the
Surface of a Zeolitic Imidazolate Framework. J. Am. Chem. Soc..

[12] Wang C., Li G., Guo H. (2022). Heterogeneous
Dimerization of Ethylene by Coordinatively
Unsaturated Metal Sites in Two Forms of Ni-MIL-77. Mol. Catal..

[13] Andrei R. D., Popa M. I., Fajula F., Hulea V. (2015). Heterogeneous Oligomerization
of Ethylene over Highly Active and Stable Ni-AlSBA-15 Mesoporous Catalysts. J. Catal..

[14] Canivet J., Aguado S., Schuurman Y., Farrusseng D. (2013). MOF-Supported
Selective Ethylene Dimerization Single-Site Catalysts through One-Pot
Postsynthetic Modification. J. Am. Chem. Soc..

[15] Metzger E.
D., Brozek C. K., Comito R. J., Dincă M. (2016). Selective
Dimerization of Ethylene to 1-Butene with a Porous Catalyst. ACS Cent. Sci..

[16] Madrahimov S. T., Gallagher J. R., Zhang G., Meinhart Z., Garibay S. J., Delferro M., Miller J. T., Farha O. K., Hupp J. T., Nguyen S. T. (2015). Gas-Phase Dimerization of Ethylene
under Mild Conditions
Catalyzed by MOF Materials Containing (Bpy)­NiII Complexes. ACS Catal..

[17] Liu J., Ye J., Li Z., Otake K., Liao Y., Peters A. W., Noh H., Truhlar D. G., Gagliardi L., Cramer C. J., Farha O. K., Hupp J. T. (2018). Beyond the Active Site: Tuning the Activity and Selectivity
of a Metal–Organic Framework-Supported Ni Catalyst for Ethylene
Dimerization. J. Am. Chem. Soc..

[18] Olivier-Bourbigou, H. ; Favre, F. ; Forestière, A. ; Hugues, F. Ionic Liquids and Catalysis: The IFP Biphasic Difasol Process. In Handbook of Green Chemistry; John Wiley & Sons, Ltd, 2010, pp. 101–126. DOI: 10.1002/9783527628698.hgc005.

[19] Kang Y.-S., Lu Y., Chen K., Zhao Y., Wang P., Sun W.-Y. (2019). Metal–Organic
Frameworks with Catalytic Centers: From Synthesis to Catalytic Application. Coord. Chem. Rev..

[20] Gao W.-Y., Cardenal A. D., Wang C.-H., Powers D. C. (2019). In Operando
Analysis
of Diffusion in Porous Metal-Organic Framework Catalysts. Chem. - Eur. J..

[21] Alzamly A., Bakiro M., Hussein Ahmed S., Siddig L. A., Nguyen H. L. (2022). Linear
α-Olefin Oligomerization and Polymerization Catalyzed by Metal-Organic
Frameworks. Coord. Chem. Rev..

[22] Agirrezabal-Telleria I., Soukri M., Lail M. A., López N., Gandarias I., Oregui-Bengoechea M., Ortuño M. A., Luz I., Luz I., Ortuño M. A. (2019). Gas Reactions under
Intrapore Condensation Regime within Tailored Metal–Organic
Framework Catalysts. Nat. Commun..

[23] Li Z., Schweitzer N. M., League A. B., Bernales V., Peters A. W., Getsoian A., Wang T. C., Miller J. T., Vjunov A., Fulton J. L. (2016). Sintering-Resistant
Single-Site Nickel Catalyst
Supported by Metal–Organic Framework. J. Am. Chem. Soc..

[24] Kømurcu M., Lazzarini A., Kaur G., Borfecchia E., Øien-Ødegaard S., Gianolio D., Bordiga S., Lillerud K. P., Olsbye U. (2021). Co-Catalyst Free Ethene Dimerization
over Zr-Based Metal-Organic Framework (UiO-67) Functionalized with
Ni and Bipyridine. Catal. Today.

[25] Alezi D., Oppenheim J. J., Sarver P. J., Iliescu A., Dinakar B., Dincă M. (2023). Tunable Low–Relative
Humidity and High–Capacity
Water Adsorption in a Bibenzotriazole Metal–Organic Framework. J. Am. Chem. Soc..

[26] Metzger E. D., Comito R. J., Hendon C. H., Dincă M. (2017). Mechanism
of Single-Site Molecule-Like Catalytic Ethylene Dimerization in Ni-MFU-4
l. J. Am. Chem. Soc..

[27] Skinner W., Bishop E., Cambour P., Fuqua S., Lim P. (1960). Studies of
the Reaction of Ethylene with Aluminum Trialkyls. Ind. Eng. Chem..

[28] Holland P. M., Eaton B. E., Hanley H. J. M. (1983). A Correlation of the Viscosity and
Thermal Conductivity Data of Gaseous and Liquid Ethylene. J. Phys. Chem. Ref. Data.

[29] Agirrezabal-Telleria I., Iglesia E. (2017). Stabilization
of Active, Selective, and Regenerable
Ni-Based Dimerization Catalysts by Condensation of Ethene Withinordered
Mesopores. J. Catal..

[30] Chen W., Elumalai P., Mamlouk H., Rentería-Gómez Á., Veeranna Y., Shetty S., Kumar D., Al-Rawashdeh M., Gupta S. S., Gutierrez O., Zhou H.-C., Madrahimov S. T. (2024). Monodentate
Phosphinoamine Nickel Complex Supported on a Metal–Organic
Framework for High-Performance Ethylene Dimerization. Adv. Sci..

[31] Song L., Chen L., Sun J., Wang L., Li M., Cai Z. (2025). MOF-Supported Diimine Nickel Catalyst for Highly Active and Selective
Ethylene Dimerization. ACS Catal..

[32] Mancuso J. L., Gaggioli C. A., Gagliardi L., Hendon C. H. (2021). Singlet-to-Triplet
Spin Transitions Facilitate Selective 1-Butene Formation during Ethylene
Dimerization in Ni­(II)-MFU-4l. J. Phys. Chem.
C.

[33] Wang C., An B., Lin W. (2019). Metal–Organic
Frameworks in Solid–Gas
Phase Catalysis. ACS Catal..

