# Supplementary material for: Selective Gas-Phase Ethylene Dimerization to 1‑Butene in a Scalable Metal–Organic Framework
Source: J Am Chem Soc. 2026 Feb 17;148(8):8832–8. doi: 10.1021/jacs.5c21528 (PMC12964411; doi:10.1021/jacs.5c21528)
Supplement: Supplementary file 1 [file ja5c21528_si_001.pdf]

Supplementary Information for

Selective Gas-Phase Ethylene Dimerization to 1-Butene in a Scalable Metal–Organic Framework

Eric You‡[a], Xiao Zhang‡[b], Patrick Sarver[a], Mircea Dincă*[b]

[a]Department of Chemistry, Massachusetts Institute of Technology, 77 Massachusetts Avenue, Cambridge, Massachusetts 02139, United States

[b]Department of Chemistry, Princeton University, Princeton, New Jersey 08544, United States

‡ E. Y. and X. Z. contributed equally to this work

*Corresponding Author: mdinca@princeton.edu

Table of contents

1 General information 2

2 Synthetic procedures 3

3 Characterization procedures of MOF 5

3.1 PXRD patterns 5

3.2 N_2 isotherms 8

3.3 SEM images 12

4 Design and construction of packed bed reactor 14

5 Gas-phase ethylene dimerization experiments with **Ni-CFA-1** 15

6 Gas Chromatography (GC) chromatograms for selected optimization experiments 18

7 Summary of homo- & heterogeneous catalysts for ethylene dimerization 26

8 Kinetic modeling calculations 28

9 References 31

1 General information

Materials: Unless otherwise noted, all materials were acquired from commercial sources and used without further purification: *N*,*N*-dimethylformamide (Millipore Sigma, 98%), *N*-methylformamide (Millipore Sigma, 99%), methanol (Millipore Sigma, 99.9%), zinc acetate dihydrate (Sigma Aldrich, 99%), nickel chloride hexahydrate (Millipore Sigma, 99.9%), nickel chloride hexahydrate (Millipore Sigma, 99.9%), 1,1'-Biphenyl-3,3'4,4'-tetraamine (AmBeed, 98%), Ethylene (99.9%, Linde), Helium (99.999%, Linde) Nitrogen (99.999%, Nitrogen), Air (Ultra Zero, Linde), Hydrogen (99.999%, Linde) , modified methylaluminoxane-12 (MMAO-12, 7 wt. % Al in toluene, Millipore Sigma), silicon carbide (325 mesh, Millipore Sigma), Quartz Wool (fine 4 μm, Technical Glass Products)

Methods:

Powder X-ray diffraction (PXRD) patterns were recorded on a Bruker Advance II diffractometer equipped with θ/2θ Bragg-Brentano geometry and Ni-filtered Cu-Kα radiation (Kα1 = 1.5406 Å). The tube voltage and current were 40 kV and 40 mA, respectively.

Nitrogen sorption isotherms were conducted on a Micromeritics ASAP 2020 Surface Area and Porosity Analyzer or Micromeritics 3Flex. Each sample was loaded in an oven-dried sample tube of known mass, activated for 16 hours overnight at 150 °C, backfilled with N_2, and weighed beforehand to determine the sample mass. The sample was placed on the analysis port of the instrument and evacuated at room temperature for 2 hours. N_2 isotherms were measured using liquid nitrogen baths (77 K). UHP grade (99.999% purity) N_2, oil-free valves and gas regulators were used for all free space corrections and measurements. BET surface area was determined using Micromeritics fitting software and MOF specific criteria.

Scanning electron microscopy (SEM) was conducted at MIT MRSEC on a Zeiss Merlin high-resolution scanning electron microscope with an InLens or High-Resolution detector at a voltage of 1–3 V and current of 100–150 pA.

^{1}H NMR spectroscopy was conducted on a Bruker Avance-III HD Nanobay spectrometer (400 MHz) or Bruker Avance Neo spectrometer (400 MHz). ^{1}H NMR spectra are internally referenced to the residual solvent signal at δ = 2.50 (DMSO-d_6) and are reported as follows: chemical shift (δ ppm), integration, and assignment. MOF samples were digested prior to ^{1}H NMR analysis by sonicating in trifluoroacetic acid, heating to 60 °C until complete dissolution (2–18 h), then diluting with DMSO-d_6 (4:1 TFA:DMSO-d_6).

Inductively coupled plasma mass spectrometry (ICP-MS) data was collected at the MIT Center for Environmental Health Sciences (CEHS) using an Agilent 7900 ICP-MS spectrometer. Calibration standards were prepared for ICP-MS analysis using analytical standard solutions purchased from VWR Chemicals BDH Aristar Plus and an aqueous 2% HNO_3 solution (prepared

from EMD Millipore OmniTrace HNO_3 and ultrafiltered water). Digestion of samples was performed in concentrated HNO_3 (67-70%, OmniTrace Ultra, EMD Millipore). Sequential dilutions were performed on digested samples and a standard curve was constructed to determine Ni and Zn loadings using data points between ICP-MS calibration standards of 1 to 1000 ppb.

Gas chromatography flame ionization detector (GC-FID) offline data was collected by a Tedlar Gas Sampling Bag (Environmental Sampling Solutions, 1L) using a gas-tight syringe (Hamilton 1700, 250 µL) to directly inject 50 µL sample into an Agilent 7890B gas chromatograph (30 meter PoraBOND Q PT capillary column) equipped with flame ionization detector. UHP grade (99.999%) He purified with an Agilent Gas Clean Filter System was used as the carrier gas at a column flow of 2.5 mL/min with a split inlet at 100:1 split ratio for total flow of 255.5 mL/min. The inlet was heated at 250 C with an initial inlet pressure of 25.918 psi. Offline samples were analyzed using the following method:oven was held at 130 °C for 3 minutes and then ramped at 20 °C/min for 6 minutes until 250 °C and held for another 4 minutes. Online GC data was collected by valve injection using a 5 µL sample loop with a Multi-gas Configuration #3 SRI 8610C gas chromatograph (MXT-Q-BOND Metal PLOT Column, 30 m, 0.53 mm ID, 20 µm, w/3.5" Coil) UHP grade (99.999%) He was used as the carrier gas at an inlet pressure of 29 psi and column flow of 20 mL/min in splitless mode. The solenoid valve was kept at 150 °C. Online samples were analyzed using the following method: oven was held at 40 °C for 1 minute and then ramped at 10 °C/min for 17 minutes until 210 °C, then ramped at 20 °C/min for 2 minute until 250 °C and held for 5 minutes, before cooling for 5 minutes back to 40 °C, for a total of 30 minutes per sample. Samples were injected by switching the valve open for 0.002 minutes at the start of each temperature program.

2 Synthetic procedures

H_2BTDD bis(1H-1,2,3-triazolo[4,5-b],[4′,5′-i])dibenzo[1,4]dioxin] (H_2BTDD) was synthesized following literature precedent.[1]

H_2bibta 5,5′-bibenzotriazole (H_2bibta) was synthesized following a modified protocol.[2]

MFU-4*l* was prepared according to the published procedure.[1] Ni-MFU-4l with the composition $Ni_{0.28}Zn_{4.72}(BTDD)_3$ was prepared by cation exchange of MFU-4*l* with $Ni(NO_3)_2$ in DMF, following previous reports.[3] To a solution of $Ni(NO_3)_2$ (1.5 g, 5.1 mmol, 21.5 equiv) in *N*,*N*-dimethylformamide (45 mL), was added MFU-4l (0.300 g, 0.24 mmol, 1 equiv) suspended in an additional 15 mL of DMF. The resulting suspension was left at room temperature for 2 days, then filtered (fine glass frit), washing extensively with *N*,*N*-dimethylformamide. The solid was suspended in *N*,*N*-dimethylformamide (40 mL), left overnight, then decanted repeating this process a total of three times. After the three washes, the solid was further suspended in 40 mL

methanol, left overnight, then decanted, repeating this process a total of three times. The resulting solid was dried under vacuum at 150 °C overnight.

CFA-1 was prepared according to the published procedure.[2] **Ni-CFA-1** of varying compositions were prepared by cation exchange of CFA-1 with either $Ni(NO_3)_2$ or $NiCl_2 \cdot 6 H_2O$ in DMF at 23 – 80 °C.

$(Zn_{0.40},Ni_{0.60})_5(OAc_{0.05},Cl_{0.95})_4(bibta)_3$: To a solution of $NiCl_2 \cdot 6 H_2O$ (9.4 g, 39.5 mmol) in *N*,*N*-dimethylformamide (200 mL) was added CFA-1 ($Zn_5(OAc)_4(bibta)_3$, 1.0 g, 0.80 mmol). The resulting suspension was stirred at 80 °C for 18 hours, then filtered (fine glass frit), washing extensively with *N*,*N*-dimethylformamide. The solid was suspended in 30 mL of DMF, left overnight, then decanted, repeating this process a total of three times. After completing the washes with DMF, the solid was resuspended in 30 mL methanol, left overnight, then decanted, repeating this process a total of three times. The solid was dried under vacuum at 150 °C overnight. The conversion of CFA-1 to **Ni-CFA-1** was determined by the molar ratios of Zn and Ni in the resultant CFA-1 by ICP-MS.

$(Zn_{0.985},Ni_{0.015})_5(OAc)_4(bibta)_3$: To a solution of $Ni(NO_3)_2 \cdot 6 H_2O$ (3.0 g, 10.3 mmol, 47.4 equiv) in *N*,*N*-dimethylformamide (30 mL), was added CFA-1 (0.250 g, 0.22 mmol, 1 equiv) suspended in an additional 5 mL of DMF. The resulting suspension was left at room temperature for 2 days, then filtered (fine glass frit), washing extensively with *N*,*N*-dimethylformamide. The solid was suspended in *N*,*N*-dimethylformamide (30 mL), left overnight, then decanted repeating this process a total of three times. After the three washes, the solid was further suspended in 30 mL methanol, left overnight, then decanted, repeating this process a total of three times. The resulting solid was dried under vacuum at 150 °C overnight. The conversion of CFA-1 to **Ni-CFA-1** was determined by the molar ratios of Zn and Ni in the resultant CFA-1 by ICP-MS.

$(Zn_{0.996},Ni_{0.0033})_5(OAc)_4(bibta)_3$: To a solution of $NiCl_2$ (1.0 mg, 4 μmol, 0.02 equiv) in DMF (5 mL) in a 20 mL vial equipped with a stir bar was added CFA-1 (0.250 g, 0.22 mmol, 1 equiv). The reaction mixture was stirred at room temperature for 1 day, filtered, and washed extensively with DMF (3 x 10 mL). The resulting solid was suspended in 30 mL of DMF, left overnight, and then decanted, repeating this process a total of three times. After the DMF washes, the solid was resuspended in 30 mL of methanol, left overnight, and then decanted, repeating this process a total of three times. The solid was dried under vacuum at 150 °C overnight to afford a light orange powder. The conversion of CFA-1 to **Ni-CFA-1** was determined by the molar ratios of Zn and Ni in the resultant CFA-1 by ICP-MS.

3 Characterization procedures of MOF

3.1 PXRD patterns

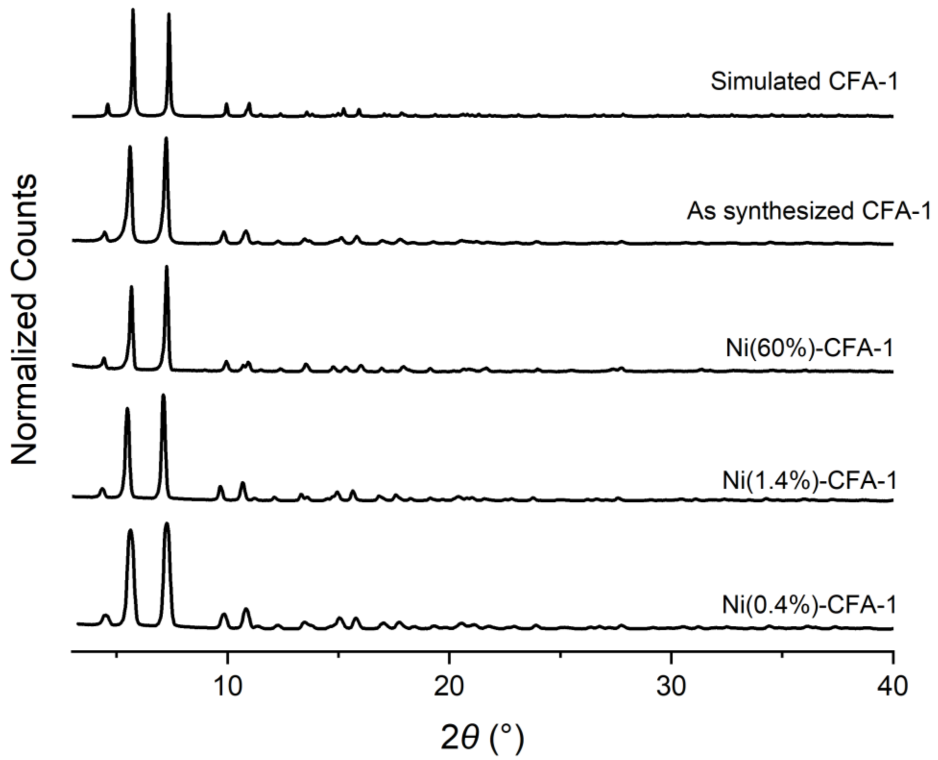

Figure S1. PXRD of CFA-1 before and after nickel cation exchange.

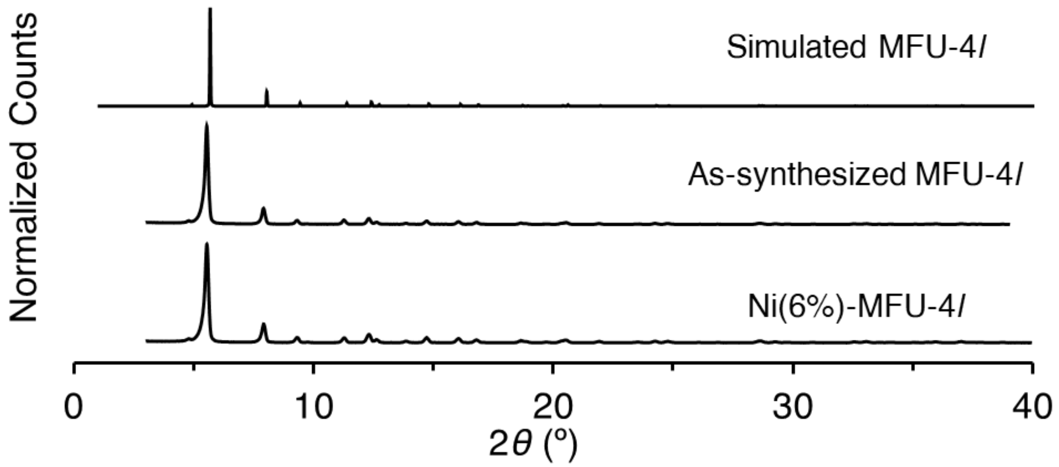

Figure S2. PXRD of MFU-4l before and after nickel cation exchange.

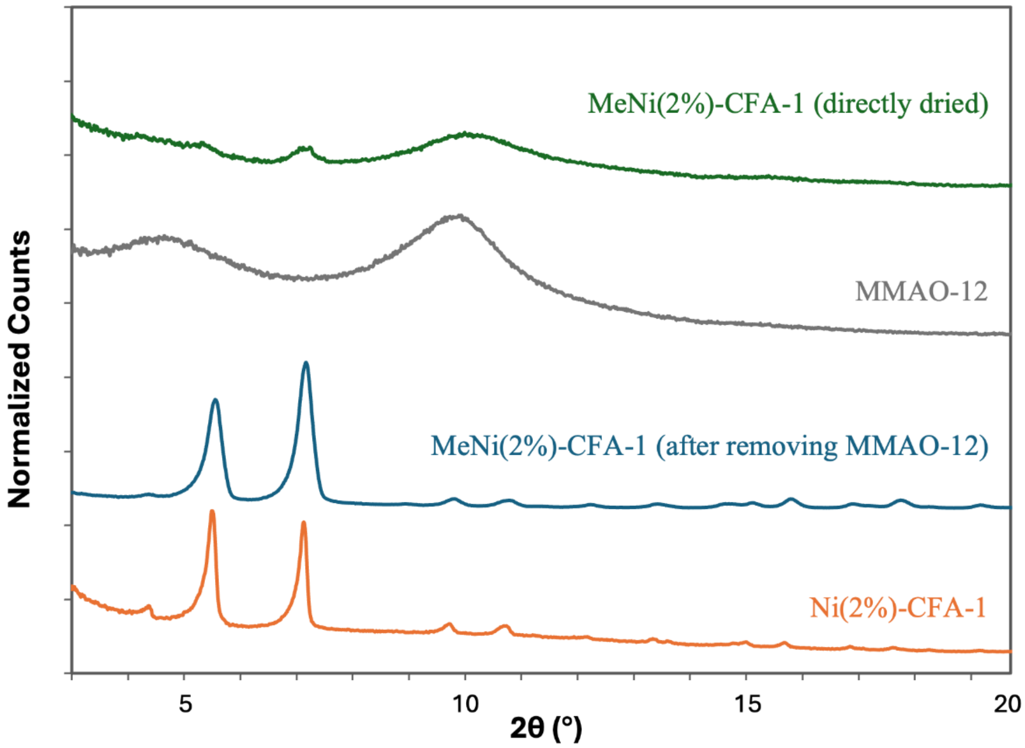

Figure S3. PXRD of **MeNi**(2%)**-CFA-1** before and after MMAO-12 activation. PXRD of the as-activated **MeNi**(2%)**-CFA-1** directly dried (in green, measured under N_2 atmosphere) shows that the PXRD pattern was mostly covered by peaks of MMAO-12 (in grey, measured under N_2 atmosphere). PXRD of the activated **MeNi**(2%)**-CFA-1** after washing with toluene and DCM to remove MMAO-12 (in blue, measured under air atmosphere) indicates that the crystallinity of **MeNi**(2%)**-CFA-1** maintained compared to the pristine **Ni**(2%)**-CFA-1** (in orange, measured under air atmosphere).

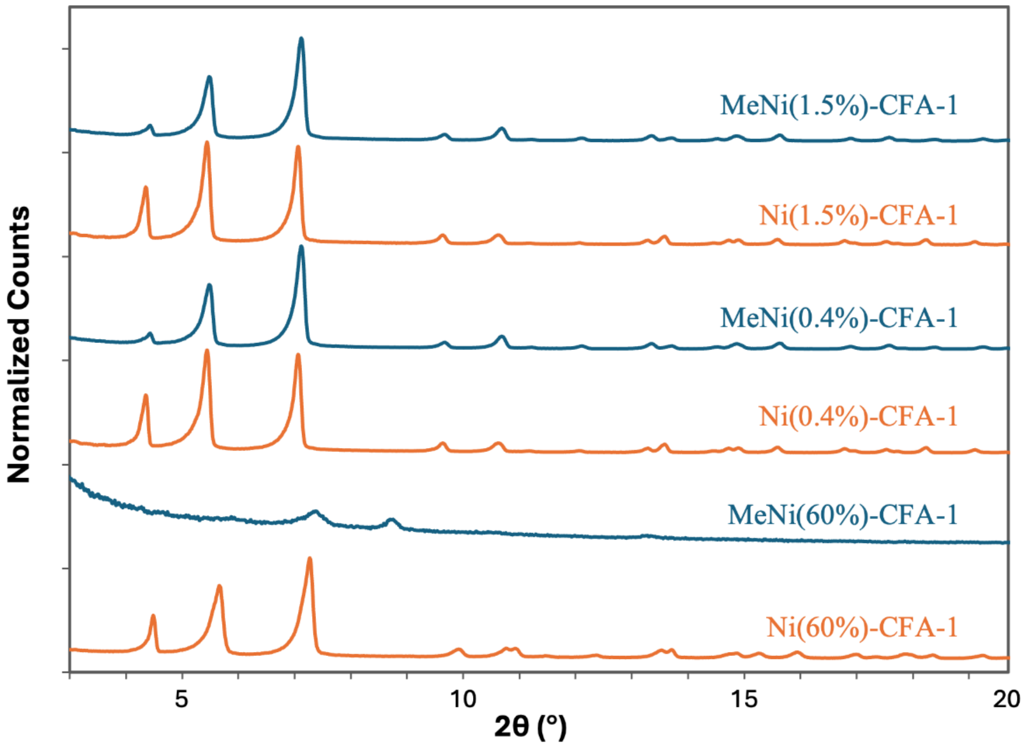

Figure S4. PXRD of **MeNi**(1.5%)**-CFA-1** compared to pristine **Ni**(1.5%)**-CFA-1**, **MeNi**(0.4%)**-CFA-1** compared to pristine **Ni**(0.4%)**-CFA-1**, **MeNi**(60%)**-CFA-1** compared to pristine **Ni**(60%)**-CFA-1**.

3.2 N_2 isotherms

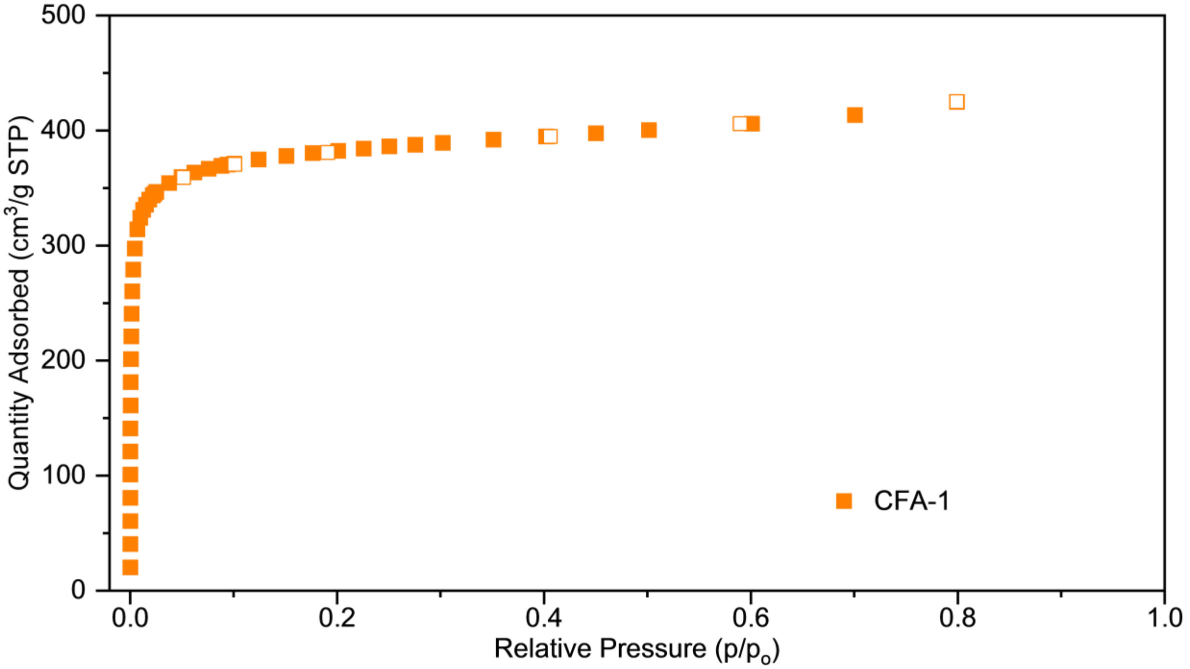

Figure S5. Isotherm for the adsorption of N_2 in CFA-1 77K. BET analysis of this isotherm gives an apparent surface area of 1512 m^2/g for CFA-1.

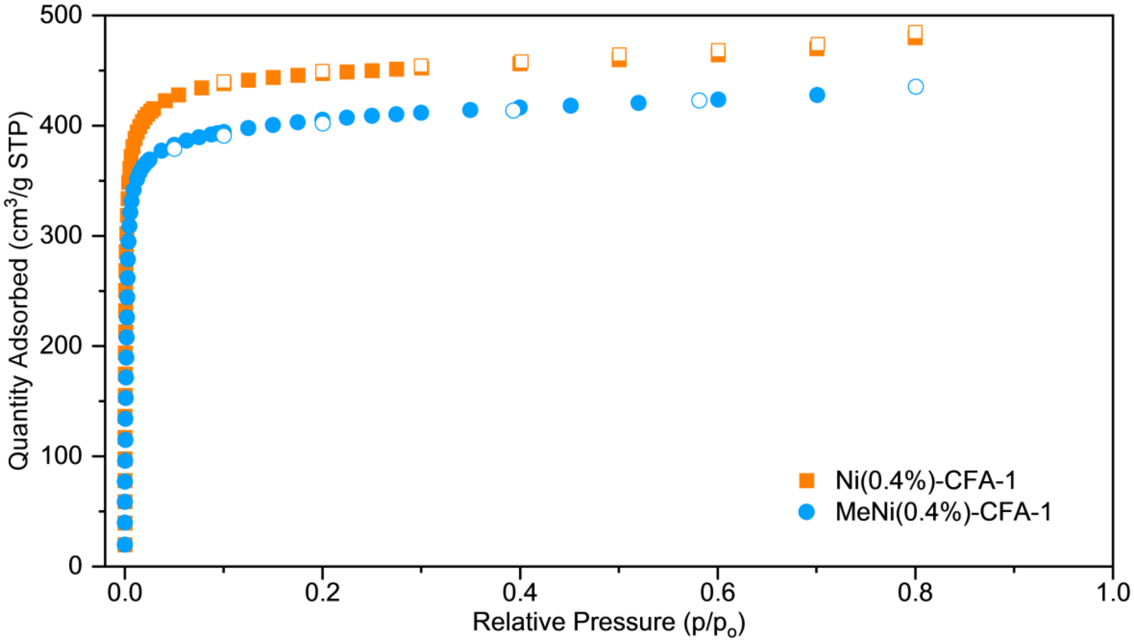

Figure S6. N_2 adsorption isotherms at 77K of pristine **Ni**(0.4%)**-CFA-1** and MMAO-12 activated **MeNi**(0.4%)**-CFA-1**. BET surface areas: **Ni**(0.4%)**-CFA-1**: 1802 m^2/g, **MeNi**(0.4%)**-CFA-1**: 1639 m^2/g.

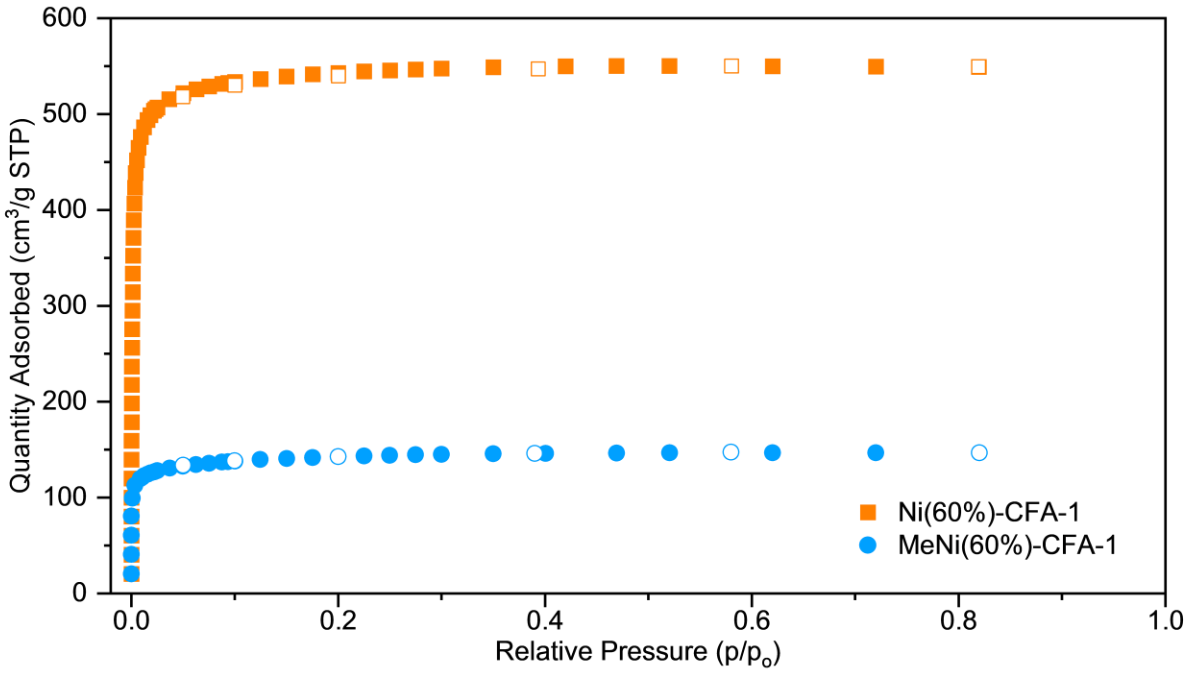

Figure S7. N_2 adsorption isotherms at 77K of pristine **Ni**(60%)**-CFA-1** and MMAO-12 activated **MeNi**(60%)**-CFA-1**. BET surface areas: **Ni**(60%)**-CFA-1**: 2212 m^2/g, **MeNi**(60%)**-CFA-1**: 553 m^2/g.

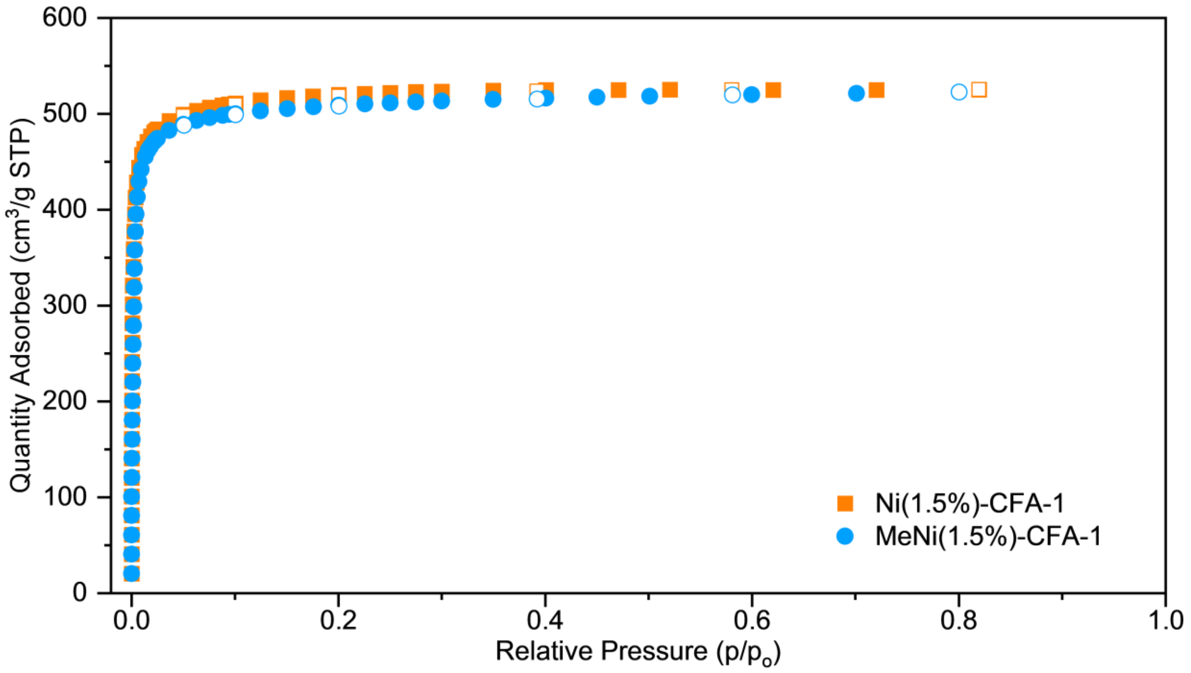

Figure S8. N_2 adsorption isotherms at 77K of pristine **Ni**(1.5%)**-CFA-1** and MMAO-12 activated **MeNi**(1.5%)**-CFA-1**. BET surface areas: **Ni**(1.5%)**-CFA-1**: 2111 m^2/g, **MeNi**(1.5%)**-CFA-1**: 2104 m^2/g.

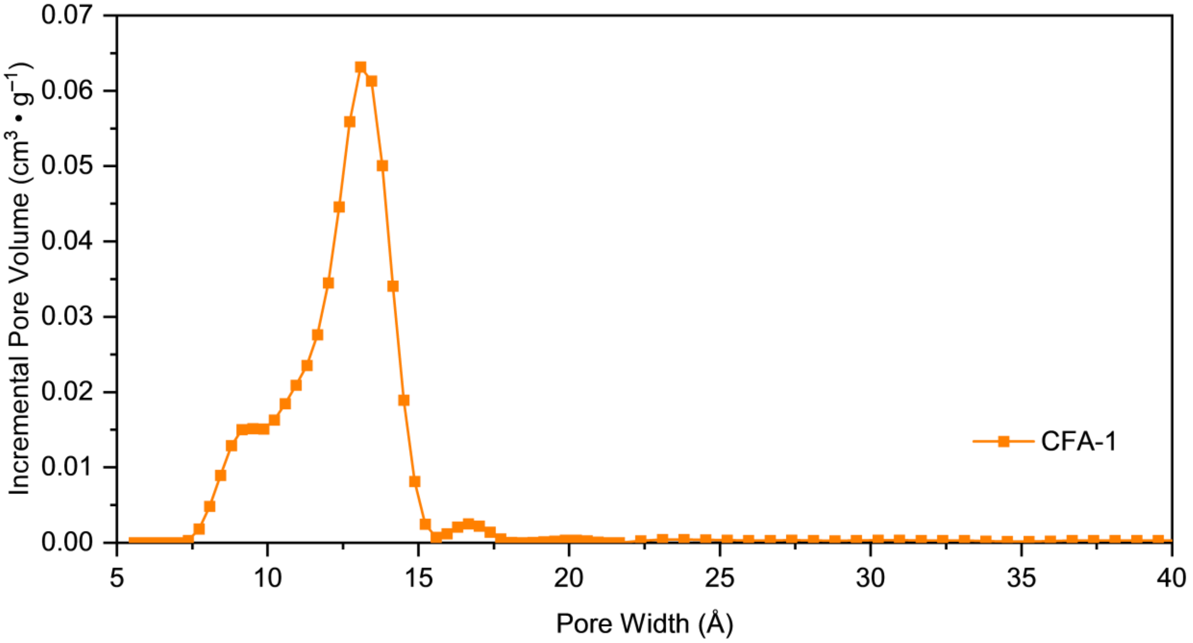

Figure S9. Pore size distribution calculated by fitting NLDFT model for pillared clay using cylindrical pore to adsorption isotherm of N_2 in CFA-1 at 77K.

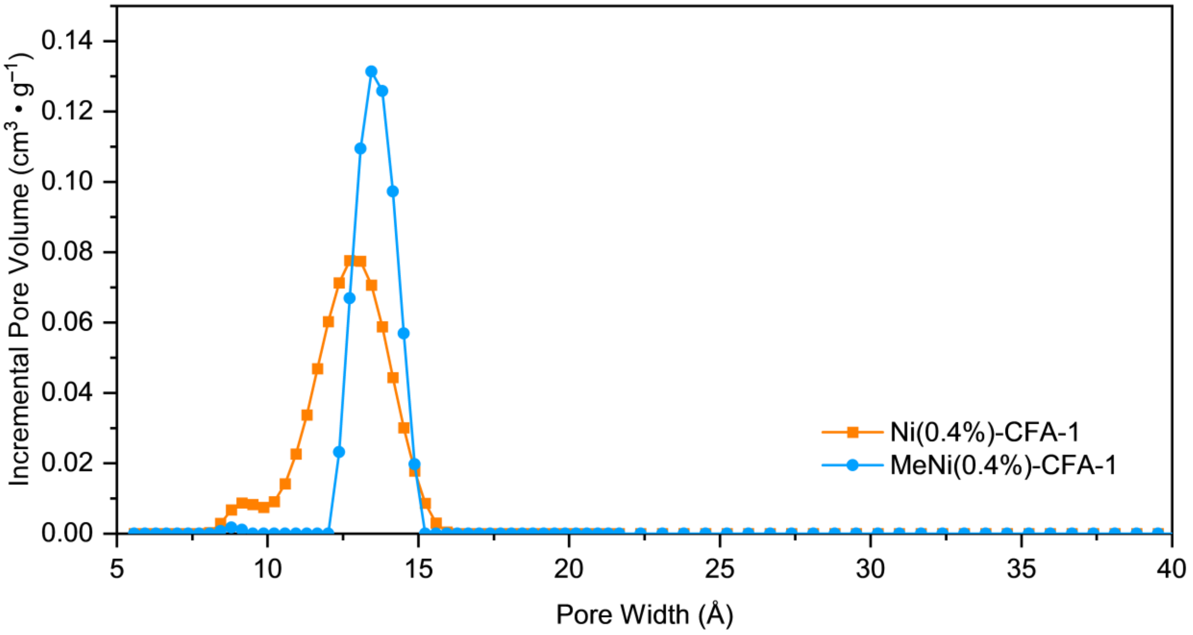

Figure S10. Pore size distribution calculated by fitting NLDFT model for pillared clay using cylindrical pore to N_2 adsorption isotherm at 77K of **Ni**(0.4%)**-CFA-1** and **MeNi**(0.4%)**-CFA-1**.

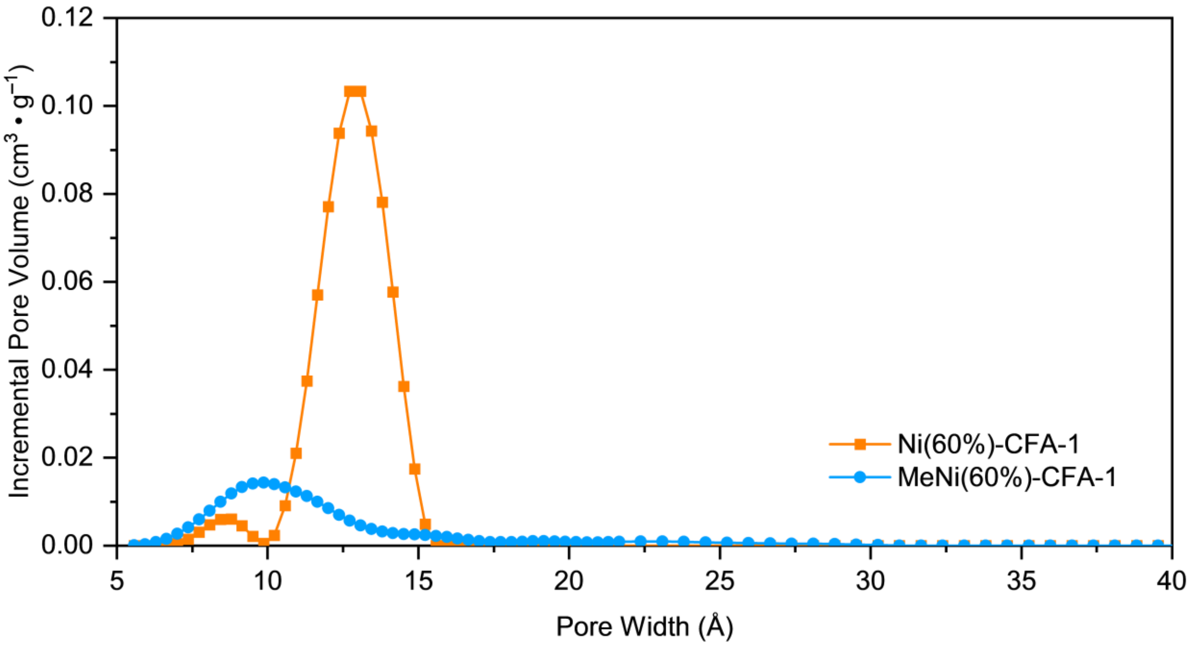

Figure S11. Pore size distribution calculated by fitting NLDFT model for pillared clay using cylindrical pore to N_2 adsorption isotherm at 77K of **Ni**(60%)**-CFA-1** and **MeNi**(60%)**-CFA-1**.

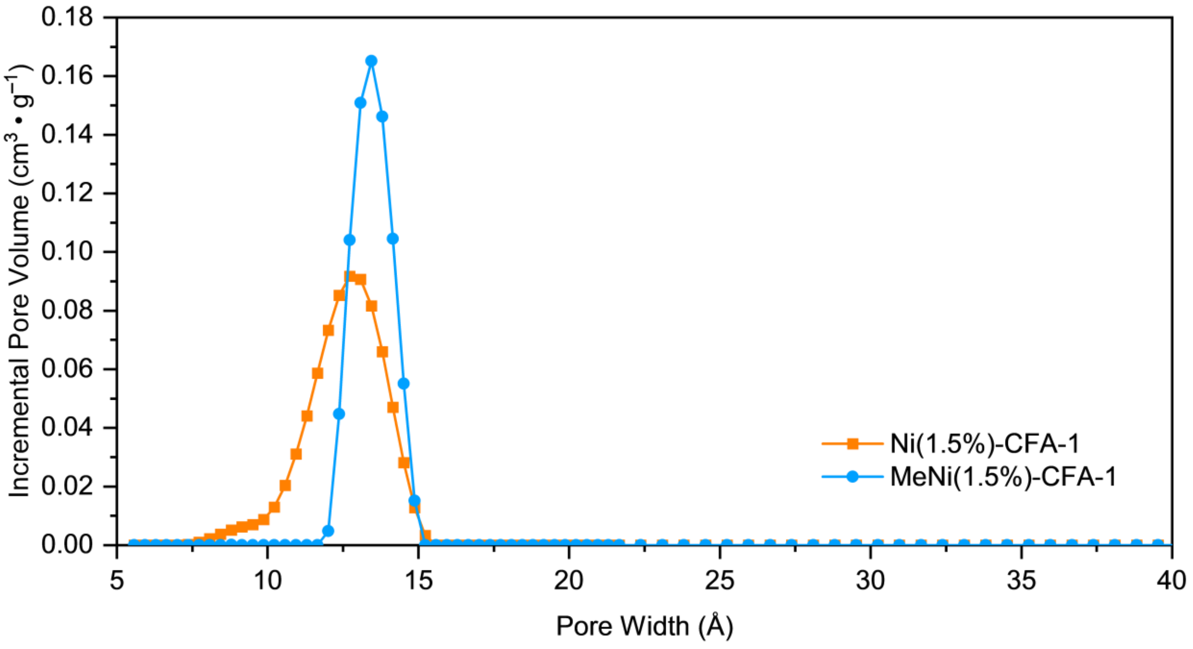

Figure S12. Pore size distribution calculated by fitting NLDFT model for pillared clay using cylindrical pore to N_2 adsorption isotherm at 77K of **Ni**(1.5%)**-CFA-1** and **MeNi**(1.5%)**-CFA-1**.

3.3 SEM images

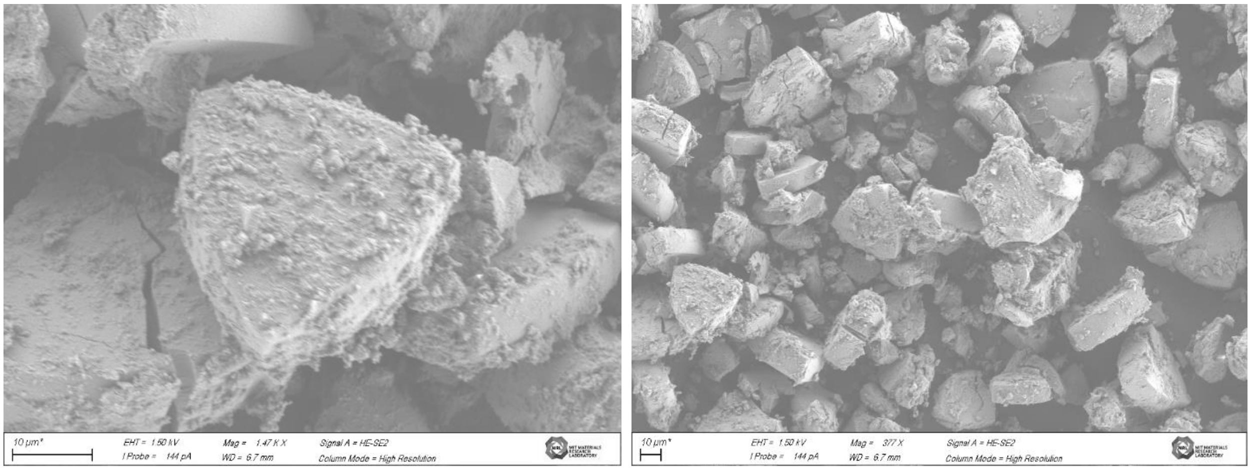

Figure S13. SEM image of CFA-1 synthesized in NMF.

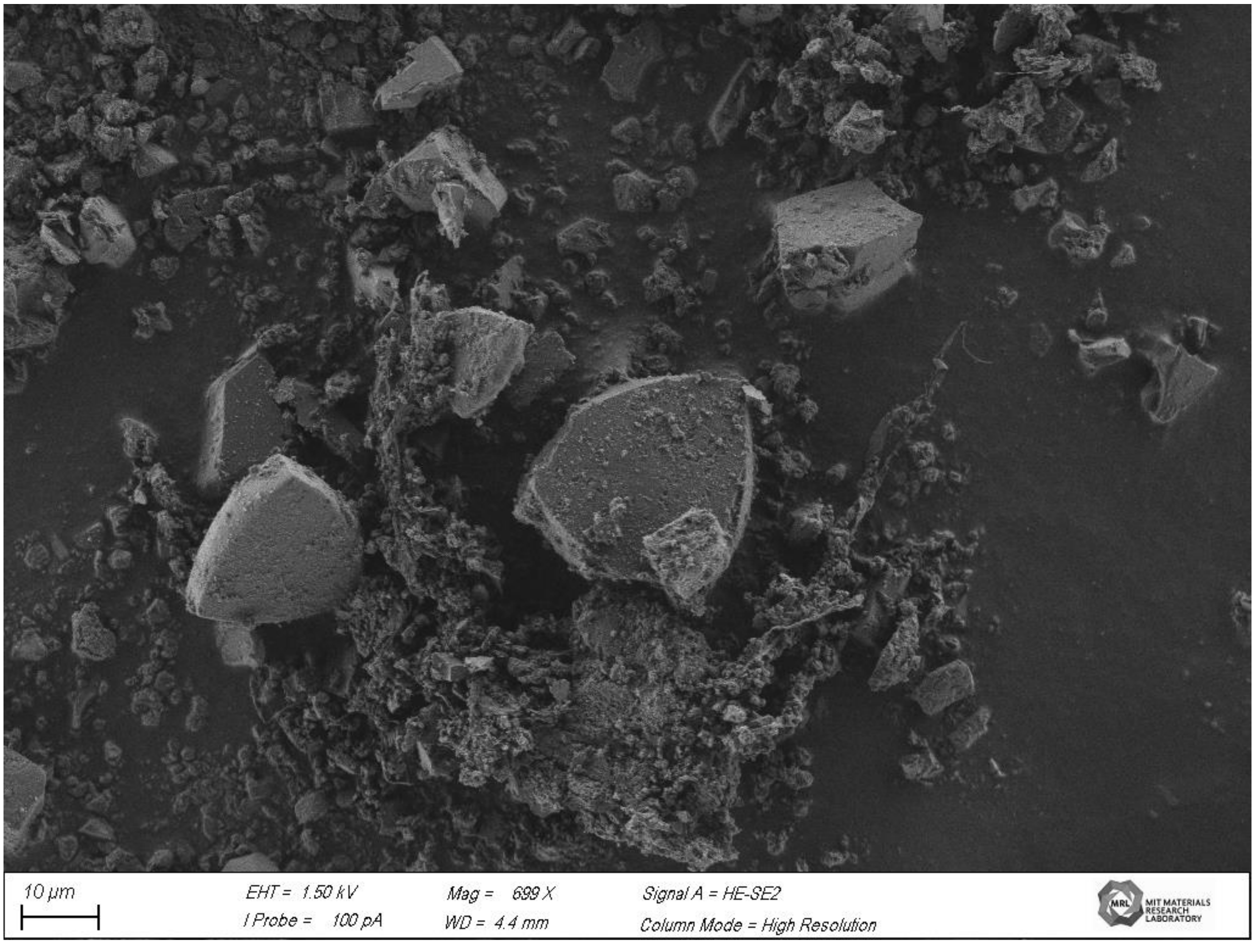

Figure S14. SEM images of **Ni**(0.4%)**-CFA-1** illustrating fragmentation of particles during nickel cation exchange with stirring.

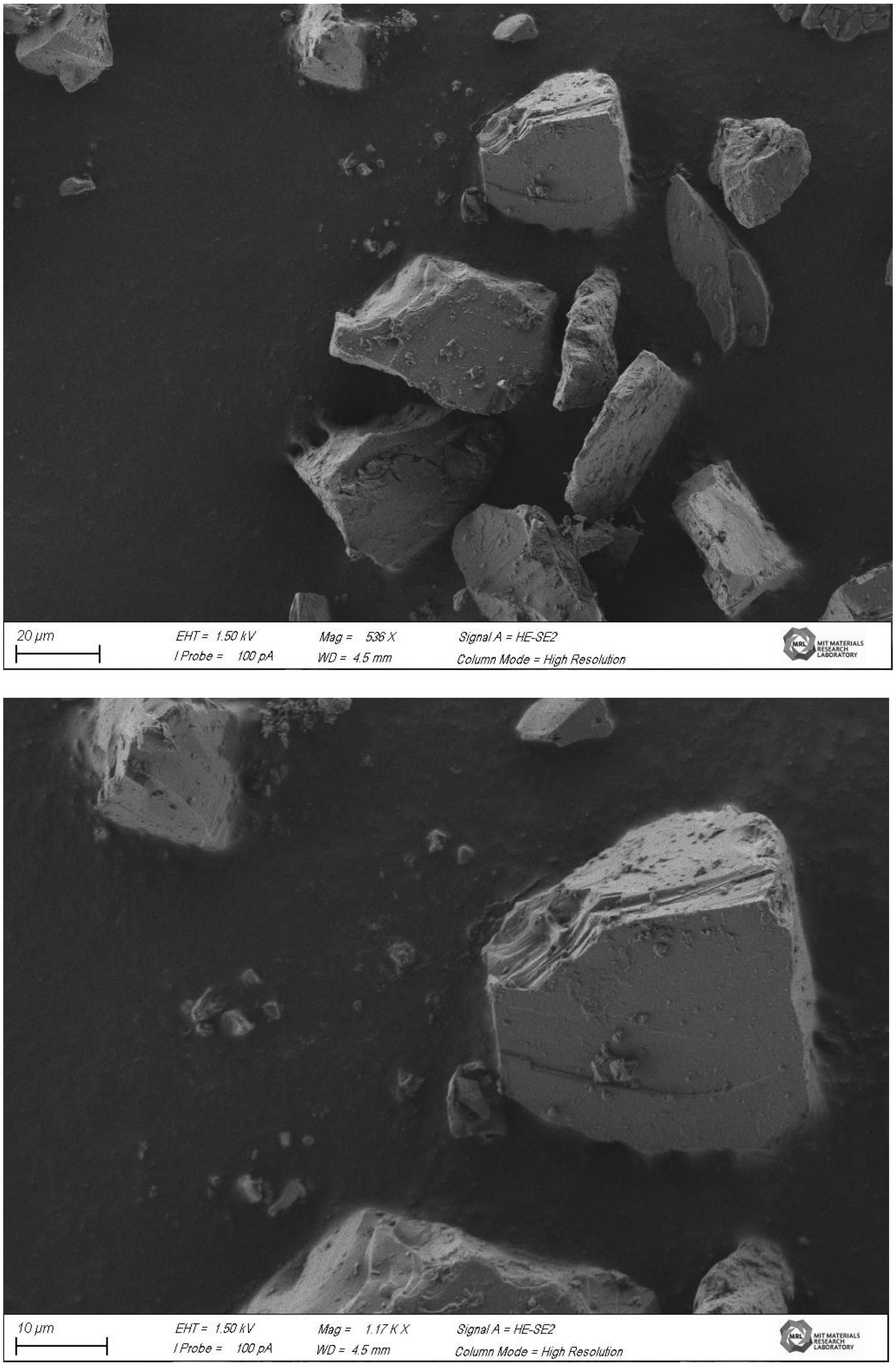

Figure S15. SEM images of catalyst reactor bed, **Ni**(0.4%)**-CFA-1** preactivated with MMAO-12, diluted with SiC (1:200 by mass), showing mostly particles of SiC and fragments of **Ni**(0.4%)**-CFA-1**.

4 Design and construction of packed bed reactor

A laboratory-scale packed-bed reactor was constructed from 1/4” and 1/8” outer diameter (OD) stainless steel tubing (McMaster); stainless steel adapters, fittings, and joints (Swagelok); two SLA5850 Mass Flow Controllers (MFC), one for ethylene and one for nitrogen (Brooks Instruments); a pressure release valve (Swagelok); and a backpressure regulator (Equilibar). Reactor temperature was monitored with a K-type thermocouple inserted just below the catalyst bed, and temperature control was enabled by jacketing the reactor with a 6” PVC pipe to which room temperature water or ice could be continuously added and removed. The reaction was conducted in a 3” long, 1/4” stainless steel tube with needle valves on either end to facilitate reaction setup under an inert environment.

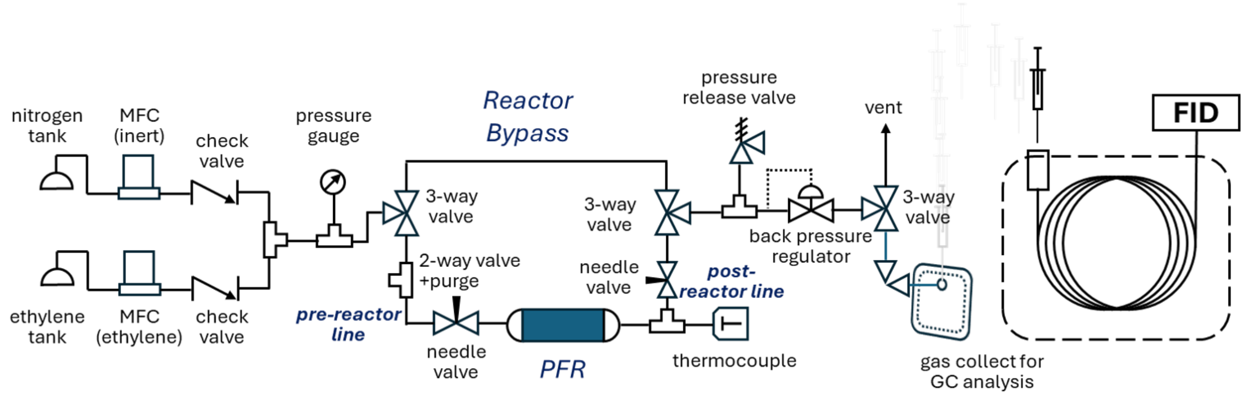

Figure S16. Schematic for reactor diagram for gas-phase ethylene dimerization showing gas flow path, data sampling, and data collection.

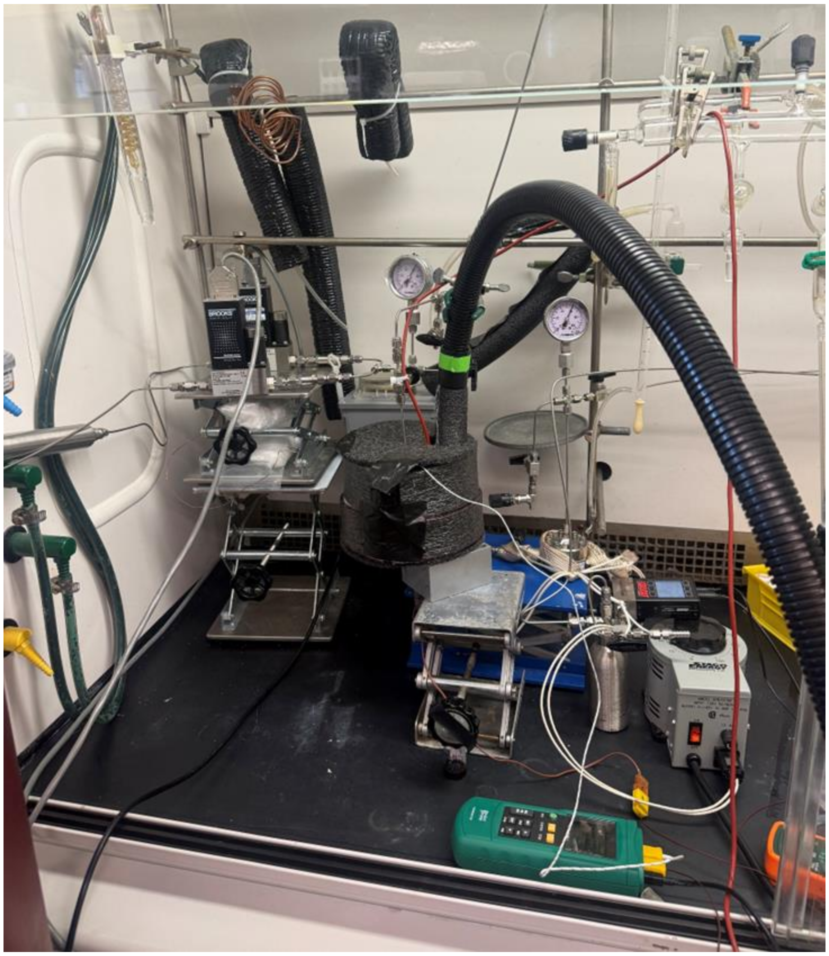

Figure S17. Image showing flow reactor setup with insulated cooling bath, immersion cooling probe, and thermocouple.

5 Gas-phase ethylene dimerization experiments with **Ni-CFA-1**

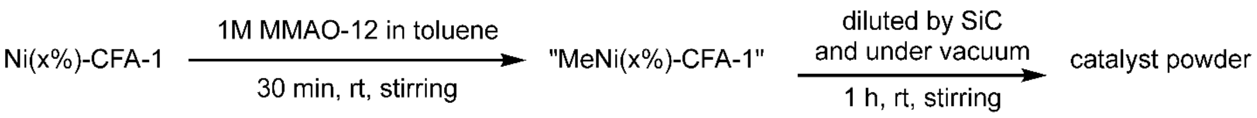

Figure S18. Preactivation of **Ni-CFA-1** and preparation of catalyst bed.

Standard Operating Procedure (Catalyst Activation, Reactor Bed Packing, and Ramp-up Procedure) A 3" long, 1/4" outer diameter (OD) straight tube reactor, packed with quartz wool 1/2" above the height of the thermometer, rubber tubing cap near the bottom of the tube, and needle valves on either end, was brought in though the large antechamber overnight.

Catalyst Activation and Reactor Bed Packing: a vial was charged with **Ni**(0.33%)-**CFA-1** (5.0 mg, 4.35 μmol), MMAO-12 (1.3 mL, 18180 equiv), stirred for 30 minutes, to which 1000 mg SiC was added and the resulting suspension was dried under vacuum for 1 hour. After drying, the final mass of solid was 1063.9 mg. The solid was gently scraped from the edges and bottom of the vial and mixed to ensure homogeneity, then 1010.8 mg (94.96% of the total mass, resulting in a catalyst loading of 4.75 mg, 4.13 μmol MOF, 0.068153 μmol Ni) was transferred to the tube reactor, topped with a plug of ½” quartz wool, and borosilicate beads to the top of the reactor tube to minimize dead volume. The top needle valve was placed back on, both needle valves were closed, and the tube reactor was removed from the glove box.

Ramp-up Procedure and Flow Experiment: In a representative ramp-up procedure and experiment, the tube reactor was installed into the flow reactor, and the space in front of the needle valves was purged with nitrogen for 20 minutes. The PVC pipe was fitted into the rubber tubing and the stainless steel strap was tightened to ensure a watertight seal and the PVC pipe was filled with water for reactions at room temperature or isopropanol for reactions at lower temperature. To ensure low temperature, the EK90 cooling coil was inserted with the thermocouple suspended next to the reactor and the temperature was set to -10 °C or other target temperature. The vent valve was closed, the top needle valve was opened, the reactor was pressurized to 60 bar of nitrogen by opening the valve on the MFC, then the bottom needle valve was opened. Flow was set to 100 mL/min ethylene by slowly increasing ethylene flow from 0 to 100 mL/min and decreasing N_2 flow from 100 mL/min to 0 mL/min by increasing/decreasing the flow rate by 20 mL/min every 10 minutes for the first 40 minutes and then increasing/decreasing the flow rate by 10 mL/min for the last 20 minutes.

Product Collection: Gas sampling bags were filled for 5 minutes in 10-minute intervals for the first hour and then every 15 minutes for the next three hours. A 250 μL syringe was rinsed with ambient air 5 times, and rinsed with gas sample from the gas bag, and then 50 μL of the gas sample from the gas bag was withdrawn and injected to the GC-MS for analysis.

Table S1. Gas phase ethylene dimerization in batch.[a]

entry	MOF	% Ni	TOF (mol/mol/h)[b]	% C_4[d]	% 1-butene[e]
1	**Ni**(5%)-**CFA-1**	5	81,500	90.4	38.7
2	**Ni**(6%)-**MFU-4*l***	6	7,400	89.2	52.6
3 (Ref. 4)	**Ni**(7.5%)-**CFA-1** (solution)	7.5	36,300	96.2	87.5
4 **(Ref.** 4)	**Ni**(1%)-**CFA-1** (solution)	1	36,100	94.9	89.9

[a]Determined by GC-FID vs. *n*-pentane standaz`rd; [b]Moles of ethylene converted per moles of nickel per hour; [c]Grams of ethylene per grams of MOF per hour; [d]Percent of oligomeric products that are C_4 olefins; [e]The overall selectivity for 1-butene among all products.

Table S2. Gas-phase ethylene dimerization with **Ni-CFA-1** in flow. [a]

entry	% Ni	reactor bed temperature (average, °C)	pressure (bar)	catalyst loading (mg)	mass velocity (g/min/g)[b]	TOF (mol/mol/h)[c]	TOF (g/g/h)[d]	conversion (%)	% C_4[e]	% α-C_4[f]	% 1-butene[g]
**1**	60%	rt[h]	50	0.5	200	7,103	516	5.4	91.8	65.8	60.3
**2**	60%	0 ℃	50	0.5	200	58,000	4213	41.4	94.4	72.8	68.5
**3**	60%	0 ℃	50	0.1	1000	70,000	5085	7.7	96.8	81.5	78.9
**4**	1.5%	rt	50	5	22.6	9,509	17	1.1	97.4	92.4	90.0
**5**	0.4%	rt	50	5	22.6	55,708	22	1.7	97.8	93.1	91.0
**6**	0.4%	rt	60	5	22.6	62,767	25	2.0	98.6	95.2	93.9
**7**	0.4%	rt	70	5	22.6	77,324	31	2.4	98.2	95.2	93.5
**8**	0.4%	0 ℃	60	5	22.6	164,330	65	5.6	98.7	96.0	94.7
**9**	0.4%	-10 °C	60	5	22.6	641,528	257	20.1	98.1	94.5	92.7
**10**	0.4%	-20 °C	60	5	22.6	275,334	110	16.3	98.2	95.2	93.5
**11**	0.4%	-10 °C	60	2	56.5	612,000	245	6.8	98.5	96.8	95.3

[a]Values determined by GC-FID, weighed average of timepoints between 2 and 4 hours following the start of ethylene flow, employing **Ni**(60%)**-CFA-1** as the catalyst, with a flow rate of 100 mL/min C_2H_4. [b]Grams of ethylene flowed per grams of catalyst per minute. [c]Moles of ethylene per mole of nickel per hour. [d]Grams of ethylene per grams of MOF per hour. [e]Percent of oligomeric products that are C_4 olefins; [f]Percent 1-butene relative to all C_4 products; [g]Overall selectivity for 1-butene among all products; [h]rt indicates that the tube was not cooled by an external bath (ambient temperature is approximately 22-25 °C).

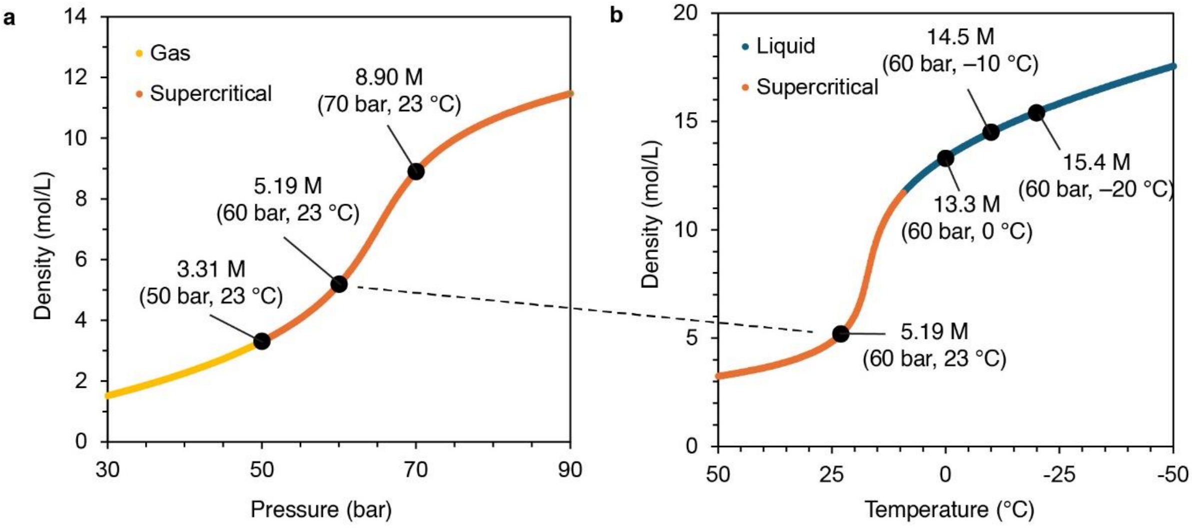

Figure S19. (a) Graph of ethylene concentrations for the optimization experiments, as indicated

by the black dots, when increasing pressure from 50 to 70 bar and (b) when decreasing temperature from 23 °C to –20 °C. Data sourced from NIST Chemistry WebBook, SRD 69.[4]

6 Gas Chromatography (GC) chromatograms for selected optimization experiments

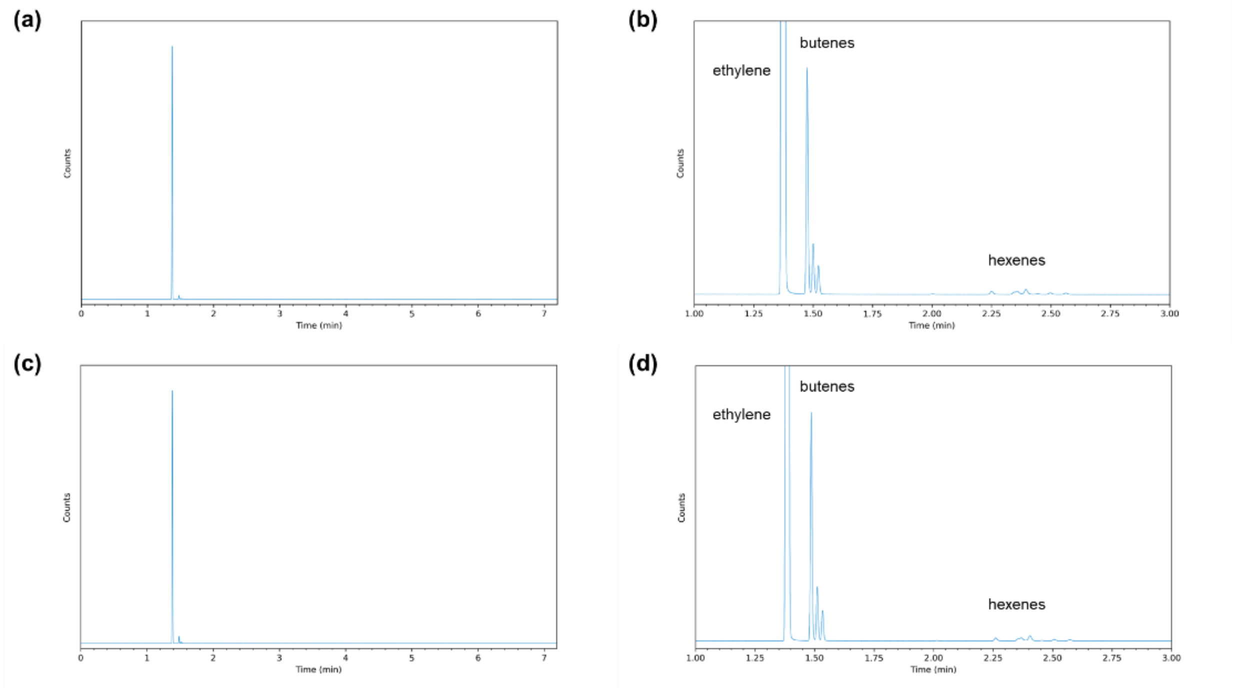

Figure S20. Chromatograms for optimization experiment in **Table S2**, Entry 1 at (a) two hours, (b) zoomed in to see the products, where butenes elute in the order 1-butene, *trans*-2-butene and *cis*-2-butene, (c) chromatogram at four hours, (d) zoomed in, to show quasi steady state conditions without significant change in conversion or selectivity.

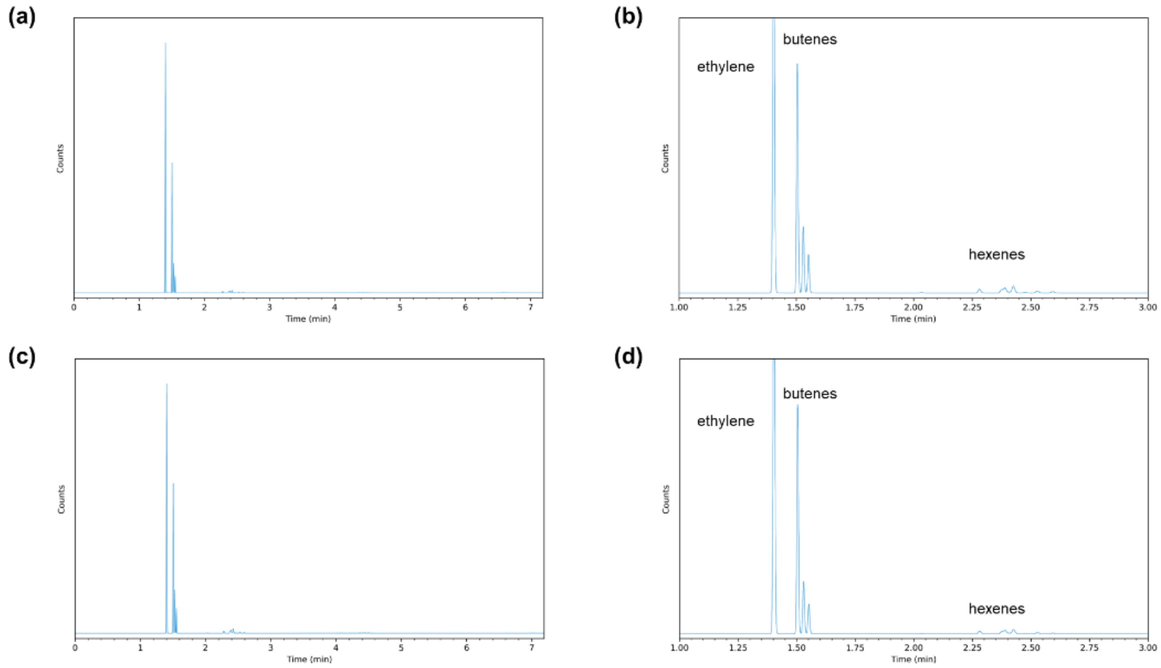

Figure S21. Chromatograms for optimization experiment in **Table S2**, Entry 2 at (a) two hours, (b) zoomed in to see the products, where butenes elute in the order 1-butene, *trans*-2-butene and *cis*-2-butene, (c) chromatogram at four hours, (d) zoomed in, to show quasi steady state conditions without significant change in conversion or selectivity.

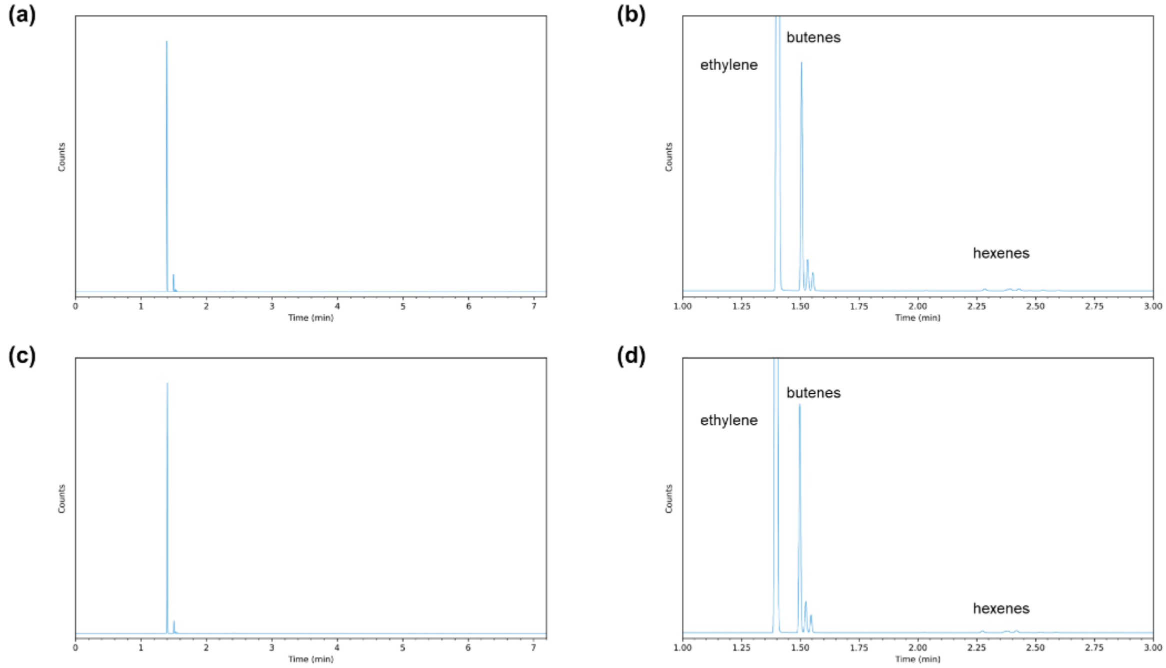

Figure S22. Chromatograms for optimization experiment in **Table S2**, Entry 3 at (a) two hours, (b) zoomed in to see the products, where butenes elute in the order 1-butene, *trans*-2-butene and

cis-2-butene, (c) chromatogram at four hours, (d) zoomed in, to show quasi steady state conditions without significant change in conversion or selectivity.

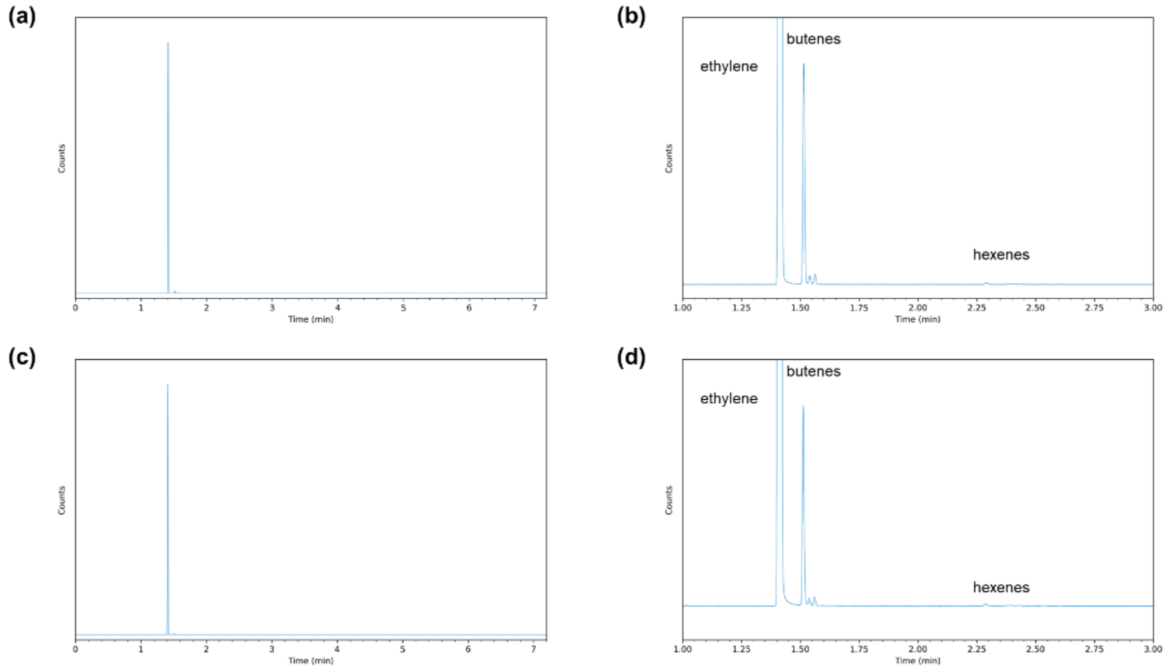

Figure S23. Chromatograms for optimization experiment in **Table S2**, Entry 4 at (a) two hours, (b) zoomed in to see the products, where butenes elute in the order 1-butene, *trans*-2-butene and *cis*-2-butene, (c) chromatogram at four hours, (d) zoomed in, to show quasi steady state conditions without significant change in conversion or selectivity.

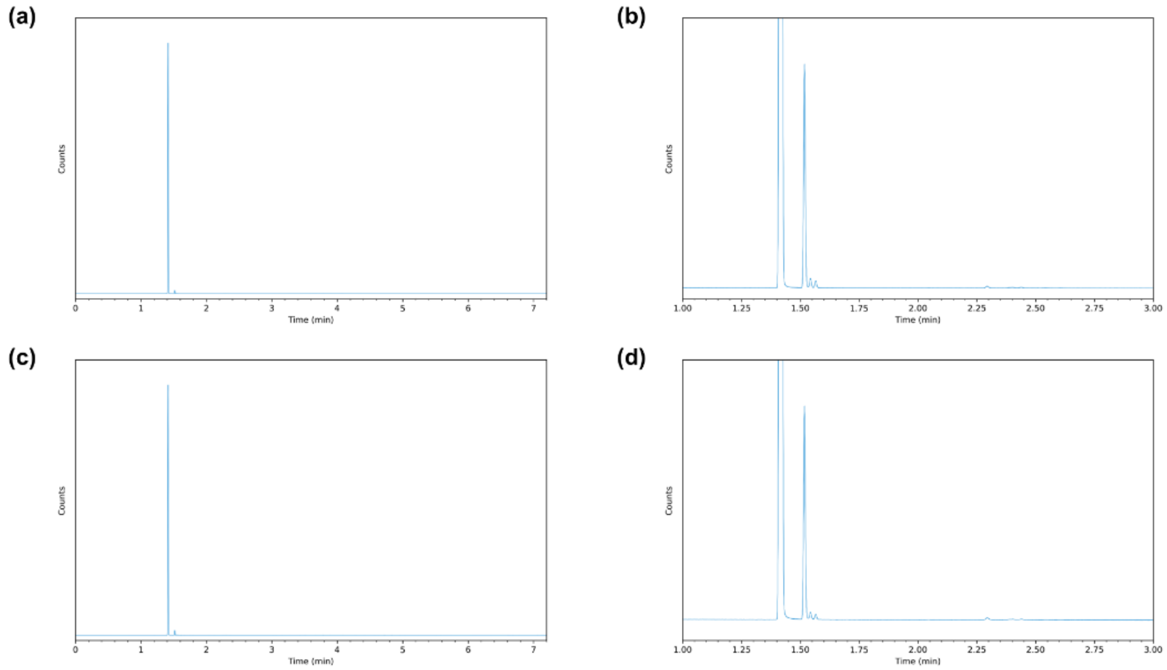

Figure S24. Chromatograms for optimization experiment in **Table S2**, Entry 5 at (a) two hours, (b) zoomed in to see the products, where butenes elute in the order 1-butene, *trans*-2-butene and *cis*-2-butene, (c) chromatogram at four hours, (d) zoomed in, to show quasi steady state conditions without significant change in conversion or selectivity.

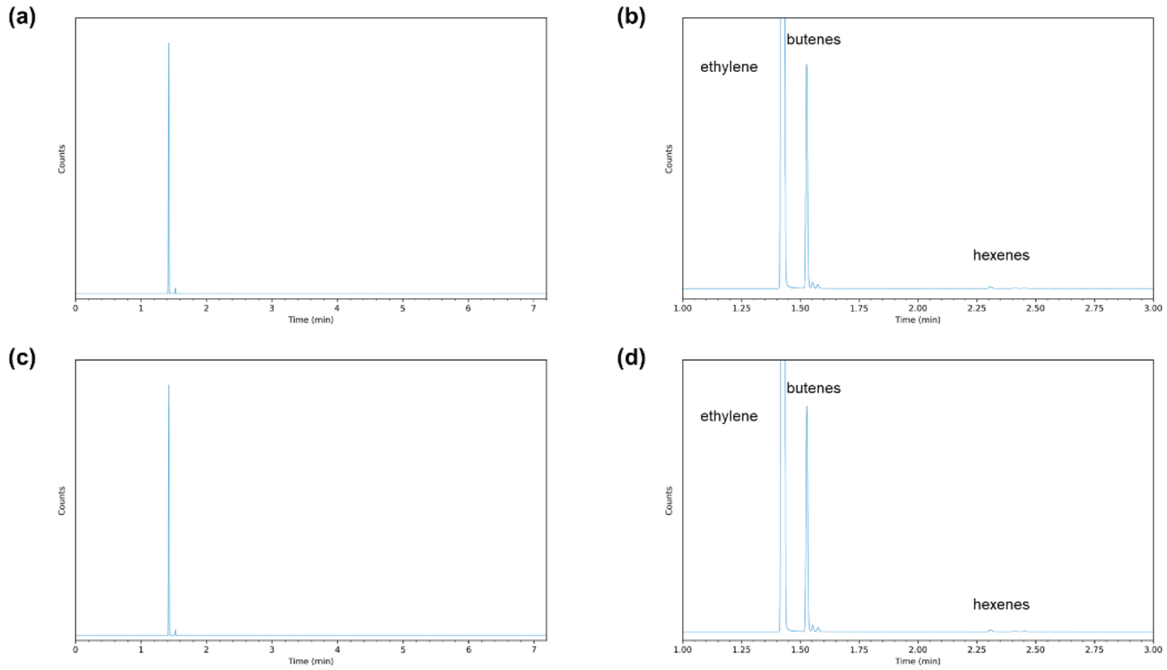

Figure S25. Chromatograms for optimization experiment in **Table S2**, Entry 6 at (a) two hours, (b) zoomed in to see the products, where butenes elute in the order 1-butene, *trans*-2-butene and

cis-2-butene, (c) chromatogram at four hours, (d) zoomed in, to show quasi steady state conditions without significant change in conversion or selectivity.

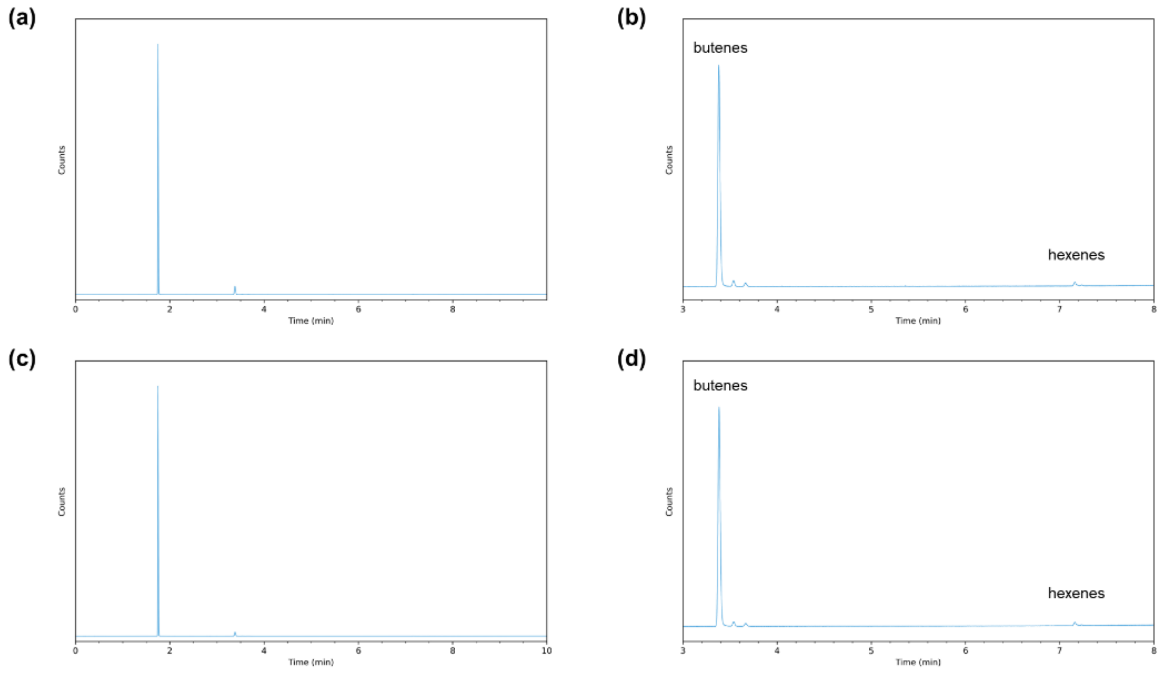

Figure S26. Chromatograms for optimization experiment in **Table S2**, Entry 7 at (a) two hours, (b) zoomed in to see the products, where butenes elute in the order 1-butene, *trans*-2-butene and *cis*-2-butene, (c) chromatogram at four hours, (d) zoomed in, to show quasi steady state conditions without significant change in conversion or selectivity.

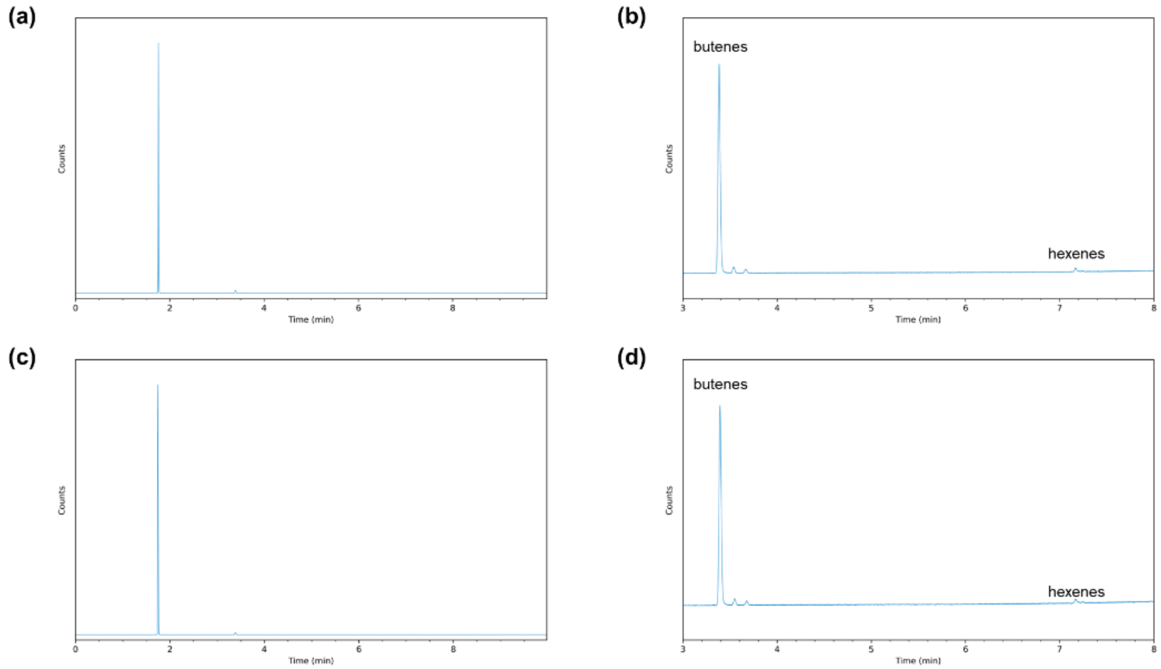

Figure S27. Chromatograms for optimization experiment in **Table S2**, Entry 8 at (a) two hours, (b) zoomed in to see the products, where butenes elute in the order 1-butene, *trans*-2-butene and *cis*-2-butene, (c) chromatogram at four hours, (d) zoomed in, to show quasi steady state conditions without significant change in conversion or selectivity.

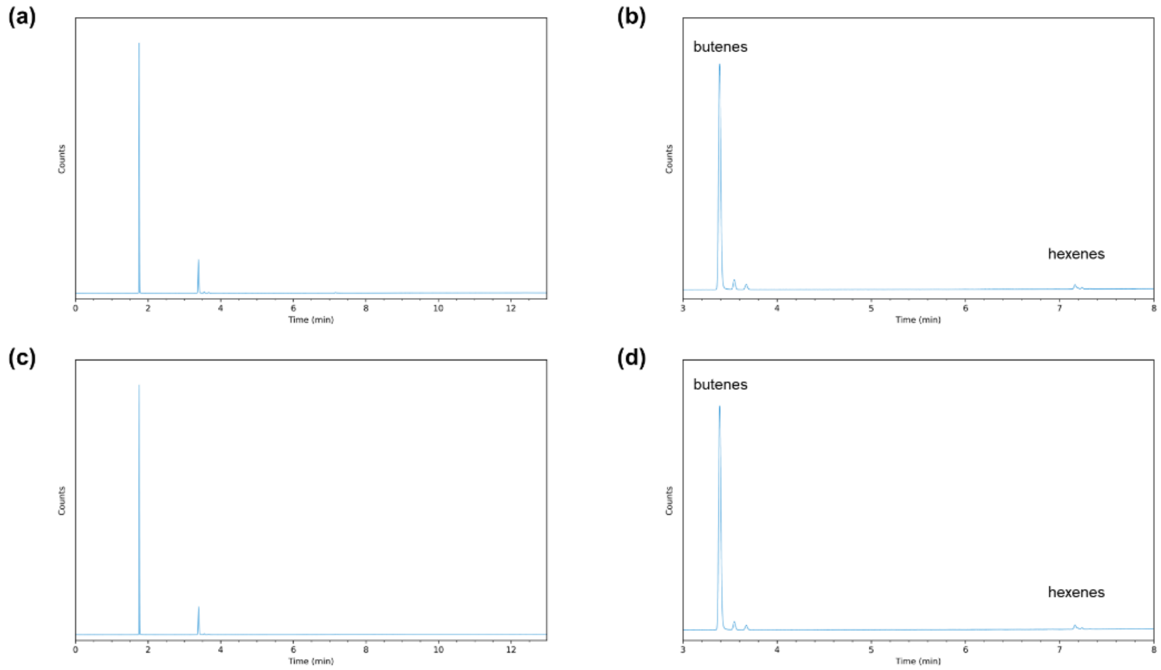

Figure S28. Chromatograms for optimization experiment in **Table S2**, Entry 9 at (a) two hours, (b) zoomed into see the products, where butenes elute in the order 1-butene, trans-2-butene and

cis-2-butene. (b) Chromatogram at four hours, (b) zoomed in, to show quasi steady state conditions without significant change in conversion or selectivity.

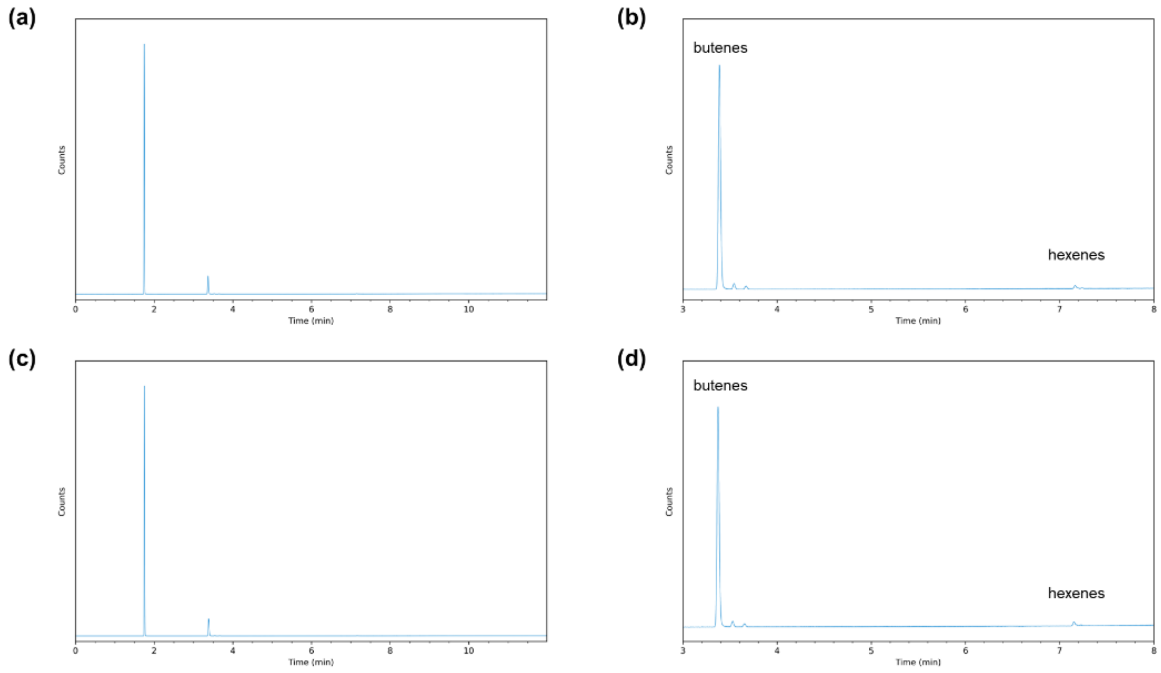

Figure S29. Chromatograms for optimization experiment in **Table S2**, Entry 10 at (a) two hours, (b) zoomed in to see the products, where butenes elute in the order 1-butene, *trans*-2-butene and *cis*-2-butene, (c) chromatogram at four hours, (d) zoomed in, to show quasi steady state conditions without significant change in conversion or selectivity.

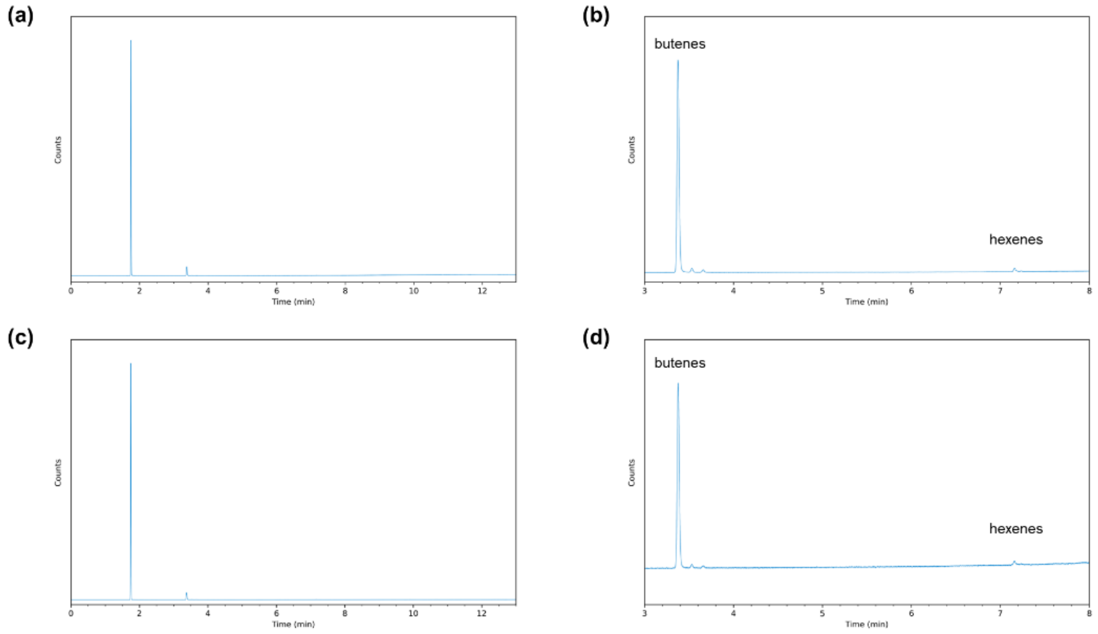

Figure S30. Chromatograms for optimization experiment in **Table S2**, Entry 11 at (a) two hours, (b) zoomed in to see the products, where butenes elute in the order 1-butene, *trans*-2-butene and *cis*-2-butene, (c) chromatogram at four hours, (d) zoomed in, to show quasi steady state conditions without significant change in conversion or selectivity.

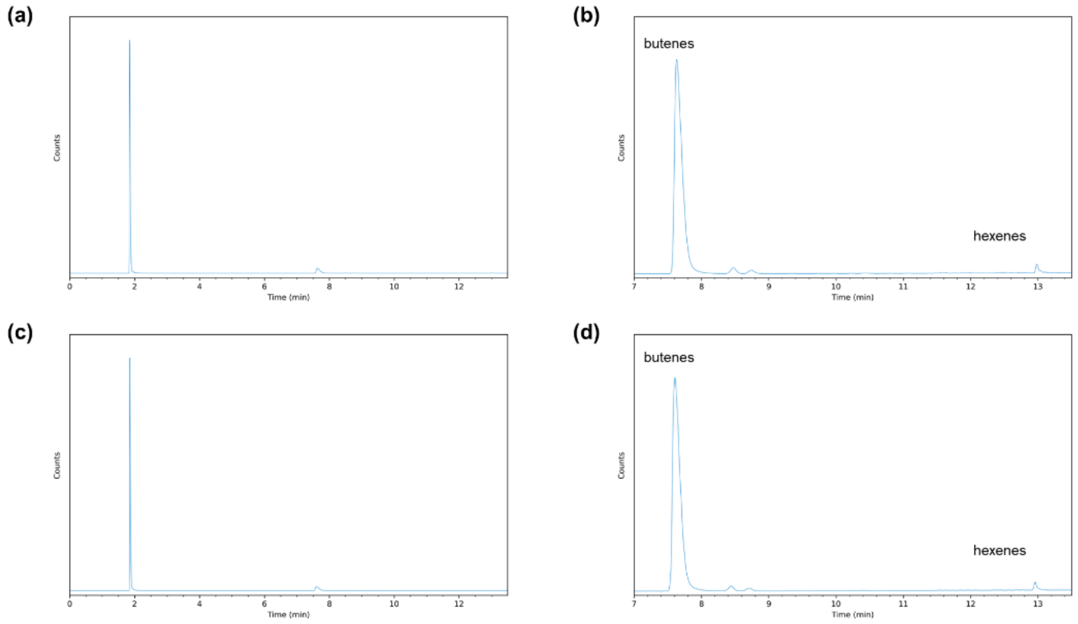

Figure S31. Chromatograms for optimization experiment in **Table S2**, Entry 12 at (a) two hours, (b) zoomed in to see the products, where butenes elute in the order 1-butene, *trans*-2-butene and *cis*-2-butene, (c) chromatogram at four hours, (d) zoomed in, to show quasi steady state conditions without significant change in conversion or selectivity.

7 Summary of homo- & heterogeneous catalysts for ethylene dimerization

Table S3. Ni-based homogeneous and heterogeneous catalysts for ethylene dimerization

Catalyst	Phase	Reactor type	P (bar)	T (°C)	TOF (mol ethylene / mol Ni / h)	%C_4[a]	%1-butene[b]	Cocat.	Eqv per Ni	Ref
$Tp^{Mes}NiCl$	Homogeneous	Batch	30	0	29,200	85	80.8	MAO	200	5
$NiBr_2$(phosphinoiminophosphorane)	Homogeneous	Batch	30	25	166,000	97.1	71.5	MAO	300	6
$NiCl_2$(methyl 2-pyridyl ketoneoxime)$_2$	Homogeneous	Batch	28	45	335,000	77	77	Et_2AlCl	150	7
$NiCl_2${N-((1-methyl-1H-imidazol-2-yl)methylene)-2-(methylthio)ethanamine}	Homogeneous	Batch	10	45	29,900	91	51	MAO	500	8
$NiBr_2${*N*-((2-phenylpyridine-6-yl)phenylmethylene)-2,6-diisopropylaniline}	Homogeneous	Batch	1.3	30	1,620,000	89	82	EASC	150	9
$NiCl_2${3,5-dimethyl-1-(3-phenoxypropyl)-1H-pyrazole}	Homogeneous	Batch	20	30	38,400	89.2	57.1	MAO	250	10
$NiCl_2$(2-thiophene aldoxime)$_2$	Homogeneous	Batch	20	45	277,143	74	74	Et_2AlCl	150	11
$NiBr_2${*N*-((4-butyl-6-phenyl pyridin-2-yl)methylene)-2,6-diisopropylaniline}	Homogeneous	Batch	5.5	30	8,308,928	83	80	EASC	150	12
$NiCl_2${2-(2-ethylquinolin-8-yl)benzoxazole}	Homogeneous	Batch	10	40	95,000	99	77.6	EASC	600	13
NiCl{*N*-[*trans*-2-(Isoindolin-2-yl)-1,2-diphenylethyl]-[3,5-bis(*tert*-butyl) salicylaldiminate]}	Homogeneous	Batch	40	30	11,700	98.5	92.1	MAO	250	14
$Ni(PPh_3)_2(C_2H_4)$–$5BF_3{\cdot}OEt_2$	Homogeneous	Batch	1	13	300	100	74.6	$BF_3{\bullet}OEt_2$	2	15
$NiBr_2$(phenoxide–iminophosphorane)	Homogeneous	Batch	30	25	72,100	97	48.1	Et_2AlCl	22.5	16
Ni(cod)(Ph_2P-NH-*i*Pr) METAMORPhos	Homogeneous	Batch	30	80	132,750	85	84	None	N/A	17
NiCl{2-(C_4H_3N-2′-CHN) C_2H_4OPh}	Homogeneous	Batch	20	30	55,900	91.5	83	MAO	250	18
$NiCl_2${2-[(2,6-difluorophenyl imino)methyl]-6-methylpyridine}	Homogeneous	Batch	2	35	53,457	100	77.8	MAO	500	19
$NiBr_2$(pyridine dicarboxamide)	Homogeneous	Batch	10	20	270,000	99	77.2	Et_2AlCl	600	20
$NiCl_2${2-phenyl-7-(2,6-diethyl-4-methylphenyl imino)-6,6-dimethylcyclopenta[b]pyridyl}	Homogeneous	Batch	5	30	206,700	98.7	89.5	MAO	2000	21
$NiBr_2$(PNSiP)	Homogeneous	Batch	10	45	427,700	88.6	59.2	Et_2AlCl	100	22
branched salicylaldimine Ni complex	Homogeneous	Batch	5	25	70,300	95.2	64	Et_2AlCl	1000	23
NiBr{2-PhO-Ph-(N=CH)-2,4-tert-butyl-2-(OC_6H_2)}	Homogeneous	Batch	20	30	59,900	97.8	89.5	MAO	300	24
$NiBr_2${2,6-diisopropylphenyl)(diphenylphosphanyl)amide}	Homogeneous	Batch	10	45	1,230,000	95.6	87.6	$EtAlCl_2$	500	25
Ni-bis-(3,5-dimethylpyrazol-1-yl)methane	Homogeneous	Batch	2.9	30	33,214	95.5	90.5	EASC	150	26

$NiBr_2${2-[bis(3,5-dimethyl pyrazol-1-yl)methyl]-8-methoxyquinoline)}	Homogeneous	Batch	2.9	30	33,558	95.9	81.4	Et_2AlCl	150	27
Ni-(Fe)MIL-101	Heterogeneous	Batch	15	10	10,400	94	95	Et_2AlCl	70	28
MixMOFs-Ni	Heterogeneous	Batch	20	40	16,400	92.7	–	Et_2AlCl	100	29
$NiCl_2$ + DEAC-MCM-41	Heterogeneous	Batch	5	30	498,000	95	2	Et_2AlCl-MCM-41	10	30
5.7%wt Ni/AlM41	Heterogeneous	Batch	35	150	15,700	58	20	None	N/A	31
Ni(1%)-MFU-4l	Heterogeneous	Batch	50	25	41,500	97.4	94.5	MAO	500	3
$Zr_6O_4(OH)_4(NiBr_2)_{0.84}(bpdc)_{5.16}$	Heterogeneous	Batch	59	55	36,000	25	–	Et_2AlCl	100	32
Ni(II)-POP-1	Heterogeneous	Batch	20	20	2,850	54	31	Et_2AlCl	69	33
Ni-UMOFNS-190	Heterogeneous	Batch	10	25	5,536	75.6	–	Et_2AlCl	500	34
**Ni**(7.5%)**-CFA-1**	Heterogeneous	Batch	50	22	37,100	95.5	91.2	MMAO-12	2000	35
Ni-ZIF-8 (0.4 wt%)	Heterogeneous	Batch	30	35	1,116,000	97	87.7	MAO	4640	36
1D-Ni-MIL-77	Heterogeneous	Batch	10	30	5,544	98	93.3	Et_2AlCl	100	37
3D-Ni-MIL-77	Heterogeneous	Batch	15	30	2,226	99.6	90	MAO	180	37
15Ni-ZIF-L	Heterogeneous	Batch	30	40	342,030	96.9	94.7	MAO	20,120	38
20Ni-MOF5 (5.32 wt%)	Heterogeneous	Batch	50	35	352,000	96.3	84.2	MAO	170	39
UiO-66-NHPiPr$_2$-$NiCl_2$	Heterogeneous	Batch	15	23	29,000	100	99	Et_2AlCl	20	40
diimine-Ni@PCN-701	Heterogeneous	Batch	20	20	43,800	98.5	97.3	$EtAlCl_2$	400	41
diimine-Ni@PCN-701	Heterogeneous	Batch	40	20	421,000	96.3	90.4	$EtAlCl_2$	320	41
H-BEA (Si/Al = 12)	Heterogeneous	Flow	26	120	132	58	–	–	–	42
NU-1000-bpy-$NiCl_2$	Heterogeneous	Flow	15	21	6,040	94	86	Et_2AlCl	70	43
Ni-AlSBA-15	Heterogeneous	Flow	35	150	14,200	53	10.6	–	–	31
5.7%wt Ni/AlM41	Heterogeneous	Flow	26	120	183	95	–	–	–	44
Ni-AIM-NU-1000	Heterogeneous	Flow	2	45	1,080	42	42	Et_2AlCl	not reported	43
Ni-MCM-41	Heterogeneous	Flow	30	-15	38,900	98	90	–	–	45
Ni-Facac-AIM-NU-1000	Heterogeneous	Flow	2	45	12.6	100	78	–	–	46
Ni-acac-AIM-NU-1000	Heterogeneous	Flow	2	45	15.84	100	82	–	–	46
Ni-UiO-66	Heterogeneous	Flow	10	200	5.4	92	45	–	–	47
UiO-66-NHPiPr$_2$-$NiCl_2$	Heterogeneous	Flow	15	23	6,000	100	99	Et_2AlCl	20	40

^{a}Percent by weight of oligomeric products that are C_4 olefins; ^{b}Overall selectivity for 1-butene by weight among all products.

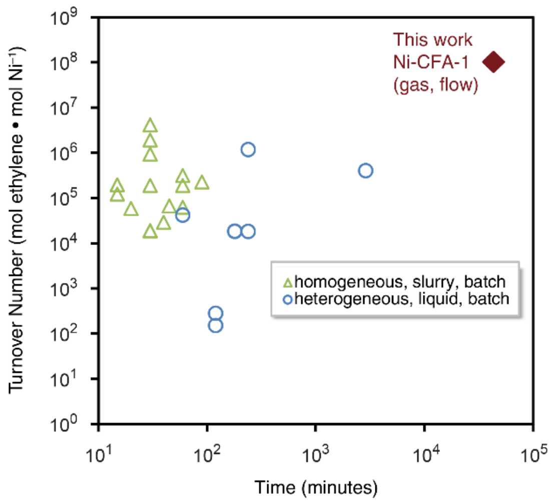

Figure S32. Comparison of the catalytic performance (reaction time and TON) of **Ni-CFA-1** in gas-phase reaction with previously reported Ni-based catalysts.

8 Kinetic modeling calculations

The kinetics model used to fit the selectivity and conversion data was derived from the assumption of a Cossee-Arlman mechanism, previously implicated for the structurally related Ni-MFU-4l.[48,49] In the Cossee-Arlman mechanism, the overall kinetics of ethylene dimerization is assumed to be first-order with respect to ethylene concentration, which is also previously shown to be true in **Ni-CFA-1** in slurry, batch conditions.[35] Rate constants for the following steps are further derived relative to the overall rate of ethylene dimerization, k_D, such that we may solve the overall ethylene rate law, as follows:

$$\frac{d[E]}{dt} = -2k_D[E]$$

$$[E]_t = [E]_0 e^{-\frac{k_D}{2}t}$$

The ethylene conversion is given by $\chi = \frac{[E]_0 - [E]_t}{[E]_0}$, allowing us to solve for a relative reaction time $\tau = t/k_D$.

$$\chi = \frac{[E]_0 - [E]_t}{[E]_0} = \left(1 - e^{-\frac{\tau}{2}}\right)$$

$$\tau = \frac{\ln(1-\chi)}{-2}$$

Next, we break down the reaction into an initial catalytic step with relative rate constants k_{A1} for the formation of 1-butene, k_{A2} for the formation of 2-butenes (cis-2-butene and trans-2-butene are lumped together for simplicity), and k_{A3} for the formation of hexenes (internal and branched hexene isomers are also grouped together for simplicity).

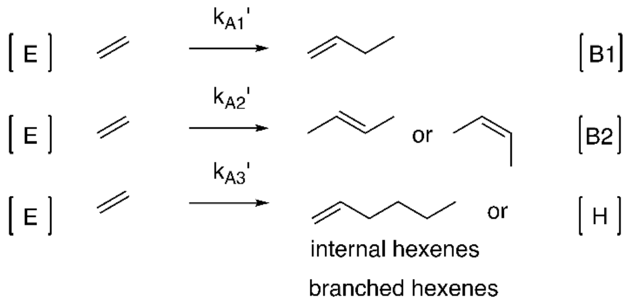

After this initial product formation and desorption, 1-butene may re-adsorb and isomerize to form the thermodynamically favored 2-butenes ($k_{B1,f}$).

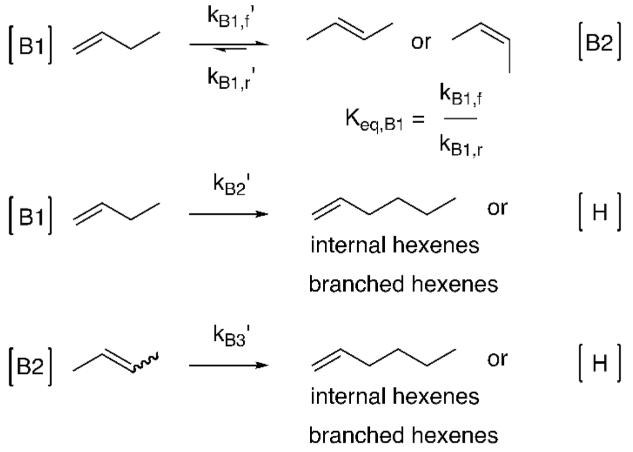

The equilibrium constant $K_{eq,B1}$ may be calculated using free energies of formation for 1-butene, cis-2-butene, and trans-2-butene.[50] At –10°C, this is given by:

$$\log K_{\frac{T}{C}} = 0.01947\left(\frac{10^3}{T}\right)^2 + 0.08663\left(\frac{10^3}{T}\right)^1 - 0.006692\ (\pm 0.008)$$

$\log K_{T/C} = .604$ for cis-2-butene ⇌trans-2-butene

$$\log K_{\frac{T}{1}} = 0.01825\left(\frac{10^3}{T}\right)^2 + 0.5326\left(\frac{10^3}{T}\right) - 0.5884(\pm 0.016)$$

$\log K_{T/1} = 1.700$ for 1-butene ⇌ trans-2-butene

$\log K_{eq,B1} = 1.398 \rightarrow K_{eq,B1} = 25.0$ for

1-butene ⇌ ½ trans-2-butene + ½ cis-2-butene

In total, the rate laws may then be written as:

$$\frac{d[E]}{dt} = -2k_d[E]$$

$$\frac{d[E]}{dt} \approx -(2k_{\mathrm{A1}} + 2k_{\mathrm{A2}} + 3k_{\mathrm{A3}})[\mathrm{E}] - k_{\mathrm{B2}}[\mathrm{B1}] - k_{B3}[B2]$$

$$\frac{d[\mathrm{B1}]}{dt} = +k_{A1}[E] - k_{\mathrm{B1,f}}[\mathrm{B1}] + \mathrm{k}_{\mathrm{B1,r}}[\mathrm{B2}] - k_{\mathrm{B2}}[\mathrm{B1}]$$

$$\frac{d[\mathrm{B2}]}{dt} = +k_{\mathrm{A2}}[E] + k_{\mathrm{B1,f}}[\mathrm{B1}] - \mathrm{k}_{\mathrm{B1,r}}[\mathrm{B2}] - k_{B3}[\mathrm{B2}]$$

$$\frac{d[\mathrm{H}]}{dt} = \mathrm{k}_{\mathrm{A3}}[E] + \mathrm{k}_{\mathrm{B2}}[\mathrm{B1}] + k_{\mathrm{B3}}[\mathrm{B2}]$$

The selectivities (by weight%) are defined as follows:

$$\%1 - \mathrm{butene} = \frac{[\mathrm{B1}]}{[\mathrm{B1}] + [\mathrm{B2}] + \frac{3}{2}[\mathrm{H}]}$$

$$\%2 - butenes = \frac{[\mathrm{B2}]}{[\mathrm{B1}] + [\mathrm{B2}] + \frac{3}{2}[H]}$$

$$\%\ \mathrm{hexenes} = \frac{[\mathrm{H}]}{\frac{2}{3}[\mathrm{B1}] + \frac{2}{3}[\mathrm{B2}] + [H]}$$

The relative rate constant estimations were performed with COPASI 4.40 software, using the Parameter Estimation module with the Levenberg – Marquardt algorithm (iteration limit of 2000, tolerance of 1e-6) to minimize the root mean squared error of estimated selectivities and conversions with the experimental selectivities and conversion until the parameters were stable and did not change within 0.00001.

9 References

(1) Denysenko, D.; Grzywa, M.; Tonigold, M.; Streppel, B.; Krkljus, I.; Hirscher, M.; Mugnaioli, E.; Kolb, U.; Hanss, J.; Volkmer, D. Elucidating Gating Effects for Hydrogen Sorption in MFU-4-Type Triazolate-Based Metal–Organic Frameworks Featuring Different Pore Sizes. *Chem. – Eur. J.* **2011**, *17* (6), 1837–1848. https://doi.org/10.1002/chem.201001872.

(2) Schmieder, P.; Denysenko, D.; Grzywa, M.; Baumgärtner, B.; Senkovska, I.; Kaskel, S.; Sastre, G.; Wüllen, L. van; Volkmer, D. CFA-1: The First Chiral Metal–Organic Framework Containing Kuratowski-Type Secondary Building Units. *Dalton Trans.* **2013**, *42* (30), 10786–10797. https://doi.org/10.1039/C3DT50787D.

(3) Metzger, E. D.; Brozek, C. K.; Comito, R. J.; Dincă, M. Selective Dimerization of Ethylene to 1-Butene with a Porous Catalyst. *ACS Cent. Sci.* **2016**, *2* (3). https://doi.org/10.1021/acscentsci.6b00012.

(4) Chase, M. W. NIST-JANAF Themochemical Tables, Fourth Edition. *J. Phys. Chem. Ref. Data, Monograph 9* **1998**, 1–1951.

(5) Kunrath, F. A.; de Souza, R. F.; Casagrande, Osvaldo L.; Brooks, N. R.; Young, V. G. Highly Selective Nickel Ethylene Oligomerization Catalysts Based on Sterically Hindered Tris(Pyrazolyl)Borate Ligands. *Organometallics* **2003**, *22* (23), 4739–4743. https://doi.org/10.1021/om034035u.

(6) Buchard, A.; Auffrant, A.; Klemps, C.; Vu-Do, L.; Boubekeur, L.; Goff, X. F. L.; Floch, P. L. Highly Efficient P–N Nickel(II) Complexes for the Dimerisation of Ethylene. *Chem. Commun.* **2007**, No. 15, 1502–1504. https://doi.org/10.1039/B618401D.

(7) Mukherjee, S.; Patel, B. A.; Bhaduri, S. Selective Ethylene Oligomerization with Nickel Oxime Complexes. *Organometallics* **2009**, *28* (10), 3074–3078. https://doi.org/10.1021/om900080h.

(8) Boudier, A.; Breuil, P.-A. R.; Magna, L.; Olivier-Bourbigou, H.; Braunstein, P. Nickel(II) Complexes with Imino-Imidazole Chelating Ligands Bearing Pendant Donor Groups (SR, OR, NR2, PR2) as Precatalysts in Ethylene Oligomerization. *J. Organomet. Chem.* **2012**, *718*, 31–37. https://doi.org/10.1016/j.jorganchem.2012.07.044.

(9) Chandran, D.; Lee, K. M.; Chang, H. C.; Song, G. Y.; Lee, J.-E.; Suh, H.; Kim, I. Ni(II) Complexes with Ligands Derived from Phenylpyridine, Active for Selective Dimerization and Trimerization of Ethylene. *J. Organomet. Chem.* **2012**, *718*, 8–13. https://doi.org/10.1016/j.jorganchem.2012.08.005.

(10) Ulbrich, A. H. D. P. S.; Campedelli, R. R.; Milani, J. L. S.; Santos, J. H. Z. dos; Casagrande, O. de L. Nickel Catalysts Based on Phenyl Ether-Pyrazol Ligands: Synthesis, XPS Study, and Use in Ethylene Oligomerization. *Appl. Catal. Gen.* **2013**, *453*, 280–286. https://doi.org/10.1016/j.apcata.2012.12.031.

(11) Tayade, K. N.; Mane, M. V.; Sen, S.; Murthy, C. N.; Tembe, G. L.; Pillai, S. M.; Vanka, K.; Mukherjee, S. A Catalytic and DFT Study of Selective Ethylene Oligomerization by Nickel(II) Oxime-Based Complexes. *J. Mol. Catal. Chem.* **2013**, *366*, 238–246. https://doi.org/10.1016/j.molcata.2012.09.029.

(12) Chandran, D.; Byeon, S. J.; Suh, H.; Kim, I. Effect of Ion-Pair Strength on Ethylene Oligomerization by Divalent Nickel Complexes. *Catal. Lett.* **2013**, *143* (7), 717–722. https://doi.org/10.1007/s10562-013-1021-7.

(13) Hao, P.; Song, S.; Xiao, T.; Li, Y.; Redshaw, C.; Sun, W.-H. Highly Active 8-Benzoxazolyl- or 8-Benzothiazolyl-2-Alkylquinolinylnickel(II) Complexes for Ethylene Dimerization and

Vinyl Polymerization of Norbornene. *Polyhedron* **2013**, *52*, 1138–1144. https://doi.org/10.1016/j.poly.2012.06.056.
(14) Wang, J.; Wan, L.; Zhang, D.; Wang, Q.; Chen, Z. Trans-1,2-Diphenylethylene Linked Isoindoline–Salicylaldiminato Nickel(II) Halide Complexes: Synthesis, Structure, Dehydrogenation, and Catalytic Activity toward Olefin Homopolymerization. *Eur. J. Inorg. Chem.* **2013**, *2013* (12), 2093–2101. https://doi.org/10.1002/ejic.201201331.
(15) Titova, Y. Y.; Belykh, L. B.; Rokhin, A. V.; Soroka, O. G.; Schmidt, F. K. Catalysis of Dimerization and Oligomerization Reactions of Lower Alkenes by Systems Based on Ni(PPh3)2(C2H4) and Ni(PPh3) n Cl (n = 2 or 3). *Kinet. Catal.* **2014**, *55* (1), 35–46. https://doi.org/10.1134/S0023158414010169.
(16) Cheisson, T.; Cao, T.-P.-A.; Le Goff, X. F.; Auffrant, A. Nickel Complexes Featuring Iminophosphorane–Phenoxide Ligands for Catalytic Ethylene Dimerization. *Organometallics* **2014**, *33* (21), 6193–6199. https://doi.org/10.1021/om500880g.
(17) Boulens, P.; Pellier, E.; Jeanneau, E.; Reek, J. N. H.; Olivier-Bourbigou, H.; Breuil, P.-A. R. Self-Assembled Organometallic Nickel Complexes as Catalysts for Selective Dimerization of Ethylene into 1-Butene. *Organometallics* **2015**, *34* (7), 1139–1142. https://doi.org/10.1021/acs.organomet.5b00055.
(18) Pinheiro, A. C.; Virgili, A. H.; Roisnel, T.; Kirillov, E.; Carpentier, J.-F.; Casagrande, O. L. Ni(II) Complexes Bearing Pyrrolide-Imine Ligands with Pendant N-, O- and S-Donor Groups: Synthesis, Structural Characterization and Use in Ethylene Oligomerization. *RSC Adv.* **2015**, *5* (111), 91524–91531. https://doi.org/10.1039/C5RA16782E.
(19) Antonov, A. A.; Semikolenova, N. V.; Talsi, E. P.; Matsko, M. A.; Zakharov, V. A.; Bryliakov, K. P. 2-Iminopyridine Nickel(II) Complexes Bearing Electron-Withdrawing Groups in the Ligand Core: Synthesis, Characterization, Ethylene Oligo- and Polymerization Behavior. *J. Organomet. Chem.* **2016**, *822*, 241–249. https://doi.org/10.1016/j.jorganchem.2016.08.031.
(20) Zhang, J.; Liu, S.; Li, A.; Ye, H.; Li, Z. Nickel(Ii) Complexes Chelated by 2,6-Pyridinedicarboxamide: Syntheses, Characterization, and Ethylene Oligomerization. *New J. Chem.* **2016**, *40* (8), 7027–7033. https://doi.org/10.1039/C6NJ00559D.
(21) Suo, H.; Zhang, Y.; Ma, Z.; Yang, W.; Flisak, Z.; Hao, X.; Hu, X.; Sun, W.-H. 2-Chloro/Phenyl-7-Arylimino-6,6-Dimethylcyclopenta*[b]*Pyridylnickel Chlorides: Synthesis, Characterization and Ethylene Oligomerization. *Catal. Commun.* **2017**, *102*, 26–30. https://doi.org/10.1016/j.catcom.2017.08.021.
(22) Huang, Y.; Zhang, L.; Wei, W.; Alam, F.; Jiang, T. Nickel-Based Ethylene Oligomerization Catalysts Supported by PNSiP Ligands. *Phosphorus Sulfur Silicon Relat. Elem.* **2018**, *193* (6), 363–368. https://doi.org/10.1080/10426507.2018.1424157.
(23) Zhang, N.; Fu, Z.; Jiang, Y.; Guo, D.; Chen, L.; Wang, J. Synthesis of Novel Structure Based Low Generation Branched Salicylaldimine Nickel Complexes and Their Catalysis of Ethylene Oligomerization. *ChemistrySelect* **2018**, *3* (37), 10428–10433. https://doi.org/10.1002/slct.201802164.
(24) de Oliveira, L. l.; da Silva, S. m.; Casagrande, A. c. a.; Stieler, R.; Casagrande Jr., O. l. Synthesis and Characterization of Ni (II) Complexes Supported by Phenoxy/Naphthoxy-Imine Ligands with Pendant N- and O-Donor Groups and Their Use in Ethylene Oligomerization. *Appl. Organomet. Chem.* **2018**, *32* (7), e4414. https://doi.org/10.1002/aoc.4414.

(25) Wei, W.; Yu, B.; Alam, F.; Huang, Y.; Cheng, S.; Jiang, T. Ethylene Oligomerization Promoted by Nickel-Based Catalysts with Silicon-Bridged Diphosphine Amine Ligands. *Transit. Met. Chem.* **2019**, *44* (2), 125–133. https://doi.org/10.1007/s11243-018-0276-7.
(26) Zubkevich, S. V.; Tuskaev, V. A.; Gagieva, S. C.; Pavlov, A. A.; Khrustalev, V. N.; Zarubin, D. N.; Kurmaev, D. A.; Kolosov, N. A.; Bulychev, B. M. Molecular Structure, Magnetic Properties and Catalytic Activity in Selective Ethylene Dimerization of Nickel (II) Complexes with Bis(3,5-Dimethylpyrazol-1-Yl)Methane. *J. Mol. Struct.* **2020**, *1206*, 127692. https://doi.org/10.1016/j.molstruc.2020.127692.
(27) Zubkevich, S. V.; Tuskaev, V. A.; Gagieva, S. Ch.; Kayda, A. S.; Khrustalev, V. N.; Pavlov, A. A.; Zarubin, D. N.; Bulychev, B. M. NNNO-Heteroscorpionate Nickel (II) and Cobalt (II) Complexes for Ethylene Oligomerization: The Unprecedented Formation of Odd Carbon Number Olefins. *Appl. Organomet. Chem.* **2020**, *34* (10), e5873. https://doi.org/10.1002/aoc.5873.
(28) Canivet, J.; Aguado, S.; Schuurman, Y.; Farrusseng, D. MOF-Supported Selective Ethylene Dimerization Single-Site Catalysts through One-Pot Postsynthetic Modification. *J. Am. Chem. Soc.* **2013**, *135* (11), 4195–4198. https://doi.org/10.1021/ja312120x.
(29) Liu, B.; Jie, S.; Bu, Z.; Li, B.-G. Postsynthetic Modification of Mixed-Linker Metal–Organic Frameworks for Ethylene Oligomerization. *RSC Adv.* **2014**, *4* (107), 62343–62346. https://doi.org/10.1039/C4RA10605A.
(30) Kermagoret, A.; Kerber, R. N.; Conley, M. P.; Callens, E.; Florian, P.; Massiot, D.; Delbecq, F.; Rozanska, X.; Copéret, C.; Sautet, P. Chlorodiethylaluminum Supported on Silica: A Dinuclear Aluminum Surface Species with Bridging M2-Cl-Ligand as a Highly Efficient Co-Catalyst for the Ni-Catalyzed Dimerization of Ethene. *J. Catal.* **2014**, *313*, 46–54. https://doi.org/10.1016/j.jcat.2014.02.006.
(31) Andrei, R. D.; Popa, M. I.; Fajula, F.; Hulea, V. Heterogeneous Oligomerization of Ethylene over Highly Active and Stable Ni-AlSBA-15 Mesoporous Catalysts. *J. Catal.* **2015**, *323*, 76–84. https://doi.org/10.1016/j.jcat.2014.12.027.
(32) I. Gonzalez, M.; Oktawiec, J.; R. Long, J. Ethylene Oligomerization in Metal–Organic Frameworks Bearing Nickel(Ii) 2,2′-Bipyridine Complexes. *Faraday Discuss.* **2017**, *201* (0), 351–367. https://doi.org/10.1039/C7FD00061H.
(33) Jeong Kim, M.; Ahn, S.; Yi, J.; T. Hupp, J.; M. Notestein, J.; K. Farha, O.; Joong Lee, S. Ni(Ii) Complex on a Bispyridine-Based Porous Organic Polymer as a Heterogeneous Catalyst for Ethylene Oligomerization. *Catal. Sci. Technol.* **2017**, *7* (19), 4351–4354. https://doi.org/10.1039/C7CY01274H.
(34) Hu, Y.; Zhang, Y.; Han, Y.; Sheng, D.; Shan, D.; Liu, X.; Cheng, A. Ultrathin Nickel-Based Metal–Organic Framework Nanosheets as Reusable Heterogeneous Catalyst for Ethylene Dimerization. *ACS Appl. Nano Mater.* **2019**, *2* (1), 136–142. https://doi.org/10.1021/acsanm.8b01762.
(35) Metzger, E. D.; Comito, R. J.; Wu, Z.; Zhang, G.; Dubey, R. C.; Xu, W.; Miller, J. T.; Dincă, M. Highly Selective Heterogeneous Ethylene Dimerization with a Scalable and Chemically Robust MOF Catalyst. *ACS Sustain. Chem. Eng.* **2019**, *7* (7), 6654–6661. https://doi.org/10.1021/acssuschemeng.8b05703.
(36) Chen, C.; Alalouni, M. R.; Dong, X.; Cao, Z.; Cheng, Q.; Zheng, L.; Meng, L.; Guan, C.; Liu, L.; Abou-Hamad, E.; Wang, J.; Shi, Z.; Huang, K.-W.; Cavallo, L.; Han, Y. Highly Active Heterogeneous Catalyst for Ethylene Dimerization Prepared by Selectively Doping Ni on the

Surface of a Zeolitic Imidazolate Framework. *J. Am. Chem. Soc.* **2021**, *143* (18), 7144–7153. https://doi.org/10.1021/jacs.1c02272.
(37) Wang, C.; Li, G.; Guo, H. Heterogeneous Dimerization of Ethylene by Coordinatively Unsaturated Metal Sites in Two Forms of Ni-MIL-77. *Mol. Catal.* **2022**, *524*, 112340. https://doi.org/10.1016/j.mcat.2022.112340.
(38) Chen, C.; Alalouni, M. R.; Xiao, P.; Li, G.; Pan, T.; Shen, J.; Cheng, Q.; Dong, X. Ni-Loaded 2D Zeolitic Imidazolate Framework as a Heterogeneous Catalyst with Highly Activity for Ethylene Dimerization. *Ind. Eng. Chem. Res.* **2022**, *61* (38), 14374–14381. https://doi.org/10.1021/acs.iecr.2c02269.
(39) Chen, C.; Meng, L.; Alalouni, M. R.; Dong, X.; Wu, Z.-P.; Zuo, S.; Zhang, H. Ultra-Highly Active Ni-Doped MOF-5 Heterogeneous Catalysts for Ethylene Dimerization. *Small* **2023**, *19* (25), 2301235. https://doi.org/10.1002/smll.202301235.
(40) Chen, W.; Elumalai, P.; Mamlouk, H.; Rentería-Gómez, Á.; Veeranna, Y.; Shetty, S.; Kumar, D.; Al-Rawashdeh, M.; Gupta, S. S.; Gutierrez, O.; Zhou, H.-C.; Madrahimov, S. T. Monodentate Phosphinoamine Nickel Complex Supported on a Metal–Organic Framework for High-Performance Ethylene Dimerization. *Adv. Sci.* **2024**, *11* (29), 2309540. https://doi.org/10.1002/advs.202309540.
(41) Song, L.; Chen, L.; Sun, J.; Wang, L.; Li, M.; Cai, Z. MOF-Supported Diimine Nickel Catalyst for Highly Active and Selective Ethylene Dimerization. *ACS Catal.* **2025**, *15* (15), 12590–12597. https://doi.org/10.1021/acscatal.5c02390.
(42) Martínez, A.; Arribas, M. A.; Concepción, P.; Moussa, S. New Bifunctional Ni–H-Beta Catalysts for the Heterogeneous Oligomerization of Ethylene. *Appl. Catal. Gen.* **2013**, *467*, 509–518. https://doi.org/10.1016/j.apcata.2013.08.021.
(43) Li, Z.; Schweitzer, N. M.; League, A. B.; Bernales, V.; Peters, A. W.; Getsoian, A. "Bean"; Wang, T. C.; Miller, J. T.; Vjunov, A.; Fulton, J. L.; Lercher, J. A.; Cramer, C. J.; Gagliardi, L.; Hupp, J. T.; Farha, O. K. Sintering-Resistant Single-Site Nickel Catalyst Supported by Metal–Organic Framework. *J. Am. Chem. Soc.* **2016**, *138* (6). https://doi.org/10.1021/jacs.5b12515.
(44) Moussa, S.; Arribas, M. A.; Concepción, P.; Martínez, A. Heterogeneous Oligomerization of Ethylene to Liquids on Bifunctional Ni-Based Catalysts: The Influence of Support Properties on Nickel Speciation and Catalytic Performance. *Catal. Today* **2016**, *277*, 78–88. https://doi.org/10.1016/j.cattod.2015.11.032.
(45) Agirrezabal-Telleria, I.; Iglesia, E. Stabilization of Active, Selective, and Regenerable Ni-Based Dimerization Catalysts by Condensation of Ethene Withinordered Mesopores. *J. Catal.* **2017**, *352*, 505–514. https://doi.org/10.1016/j.jcat.2017.06.025.
(46) Liu, J.; Ye, J.; Li, Z.; Otake, K.; Liao, Y.; Peters, A. W.; Noh, H.; Truhlar, D. G.; Gagliardi, L.; Cramer, C. J.; Farha, O. K.; Hupp, J. T. Beyond the Active Site: Tuning the Activity and Selectivity of a Metal–Organic Framework-Supported Ni Catalyst for Ethylene Dimerization. *J. Am. Chem. Soc.* **2018**, *140* (36), 11174–11178. https://doi.org/10.1021/jacs.8b06006.
(47) Yeh, B.; Vicchio, S. P.; Chheda, S.; Zheng, J.; Schmid, J.; Löbbert, L.; Bermejo-Deval, R.; Gutiérrez, O. Y.; Lercher, J. A.; Lu, C. C.; Neurock, M.; Getman, R. B.; Gagliardi, L.; Bhan, A. Site Densities, Rates, and Mechanism of Stable Ni/UiO-66 Ethylene Oligomerization Catalysts. *J. Am. Chem. Soc.* **2021**, *143* (48), 20274–20280. https://doi.org/10.1021/jacs.1c09320.

(48) Metzger, E. D.; Comito, R. J.; Hendon, C. H.; Dincă, M. Mechanism of Single-Site Molecule-Like Catalytic Ethylene Dimerization in Ni-MFU-4 *l. J. Am. Chem. Soc.* **2017**, *139* (2), 757–762. https://doi.org/10.1021/jacs.6b10300.
(49) Mancuso, J. L.; Gaggioli, C. A.; Gagliardi, L.; Hendon, C. H. Singlet-to-Triplet Spin Transitions Facilitate Selective 1-Butene Formation during Ethylene Dimerization in Ni(II)-MFU-4l. *J. Phys. Chem. C* **2021**, *125* (40), 22036–22043. https://doi.org/10.1021/acs.jpcc.1c07658.
(50) Meyer, E. F.; Stroz, D. G. Thermodynamics of N-Butene Isomerization. *J. Am. Chem. Soc.* **1972**, *94* (18), 6344–6347. https://doi.org/10.1021/ja00773a015.
